# Supplementary material for: Cell‐specific responses to the cytokine TGFβ are determined by variability in protein levels
Source: Mol Syst Biol. 2018 Jan 25;14(1):e7733. doi: 10.15252/msb.20177733 (PMC5787704; doi:10.15252/msb.20177733)
Supplement: Supplementary file 1 — Appendix [file MSB-14-e7733-s001.pdf]

Appendix

Table of content

Appendix Figure S1. Automated image analysis provides robust measurements of SMAD signaling activity 2
Appendix Figure S2. Dynamics and variability of SMAD4 signaling in single cells 3
Appendix Figure S3. Performance and robustness of constrained dynamic time warping 5
Appendix Figure S4. Parameter distributions from 30 independent population average model fits with similar goodness-of-fit 7
Appendix Figure S5. Distributions of parameter values for 30 independent fits to the signaling class dynamics with similar goodness-of-fit 8
Appendix Figure S6. Distributions of signaling protein concentrations in an artificial cell population 9
Appendix Figure S7. TGFb receptor levels are decreased in SMAD7 knock-out cells 10

I. LIST OF TIME-LAPSE MICROSCOPY EXPERIMENTS 11

Appendix Table S1: List of time-lapse microscopy experiments and the number of tracked cells per condition. 11

II. SINGLE CELL DATA ANALYSIS 13

II.A Image analysis and cell tracking 13
II.B Data preprocessing 14
II.C Measuring cell-to-cell dissimilarity using Dynamic Time Warping 14
II.D Clustering of single cell trajectories 17
II.E Local cell density 18
II.F Motility 19
II.G Sister cell analysis 19
II.H Mapping single cell trajectories to signaling classes 20
II.I TGFβ measurement 20

III. POPULATION-AVERAGE MODEL OF THE SMAD SIGNALING PATHWAY 22

III.A. Model topology 22
III.B Model equations 25
Appendix Table S2: List of model species 27
Appendix Table S3: List of model parameters 28
III.C. Model simulation 30
III.D Model calibration 33
Appendix Table S4: Datasets used for calibration of population-average model 34
Appendix Table S5A: Best-fit model parameters of the population-average model 37
Appendix Table S5B: Offsets, scaling factors and error parameters of the best-fit model 38
III.E. Model validation 39
Table S6: Datasets used for validation of population-average model 39

IV. QUANTITATIVE MODELING OF SIGNALING HETEROGENEITY 41

IV.A Derivation of six sub-population models 41
Appendix Table S7: Best-fit parameter values of the six sub population models. 42
IV.B Simulation of a complete cell population (cluster model approach) 43
IV.C Simulation of a complete cell population (simple ensemble model approach) 46
IV.D Quantitative analysis of signaling dynamics in SMAD7 knockout cells 47

V. REFERENCES 50

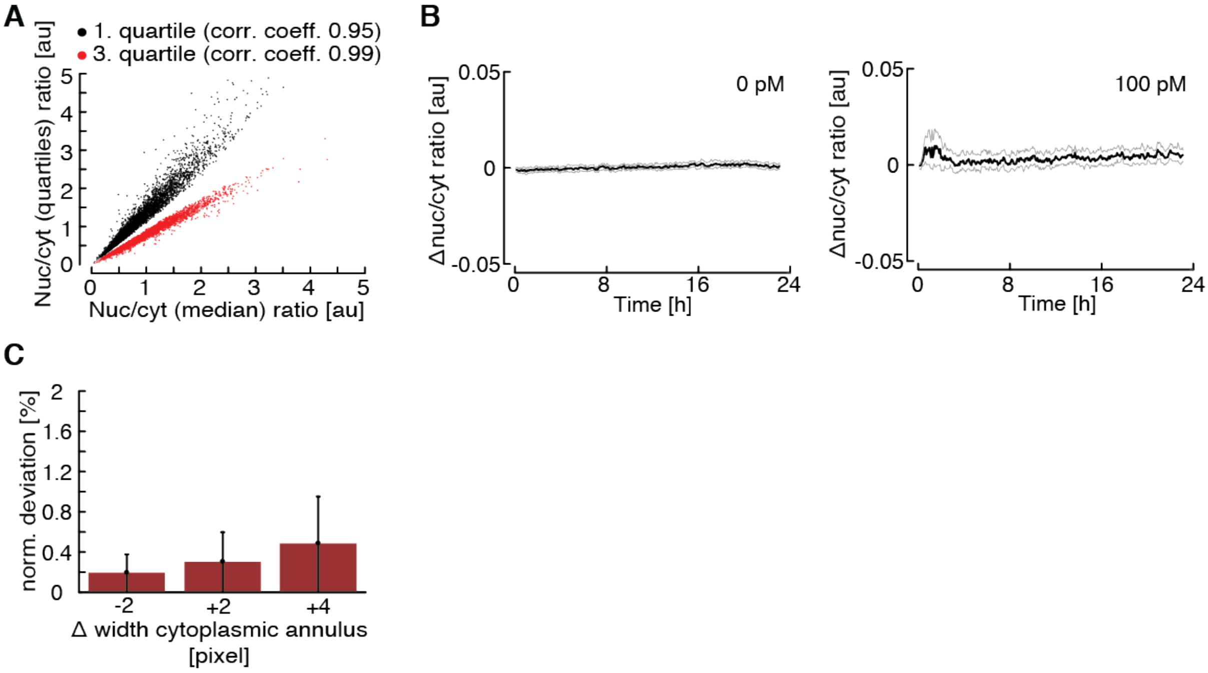

Appendix Figure S1. Automated image analysis provides robust measurements of SMAD signaling activity

(A) Calculation of nuclear to cytoplasmic SMAD2 ratios are robust to alternative measures of cytoplasmic levels. Nuc/cyt SMAD2 ratios were calculated using the median, the first or the third quartile of the fluorescence intensity in the estimated cytoplasmic region around the segmented nucleus. Graphical comparisons of the different measures as well as correlation coefficients are shown. While absolute values vary, measures are linearly correlated.

(B-C) Measured ratios are independent of the size of the estimated cytoplasmic region. Reducing the width of the cytoplasmic annulus by 2 pixels resulted in only small shifts in the nuc/cyt SMAD2 ratio (B). Differences in nuc/cyt ratios of tracked cells between quantifications with reference cytoplasmic annulus width and an adjustment of +2 pixels were compared using permutation testing; graphs indicate estimated effect sizes and corresponding 0.05-0.95% confidence intervals for stimulation with 0pM and 100pM TGFb. Deviation increased slightly with increasing stimulus strength due to the increasing dynamic range of measurements. The normalized deviation for increased or decreased width of the annulus remained below 1% (C). Bars indicate estimated effect sizes; error bars the corresponding 0.05-0.95% confidence intervals.

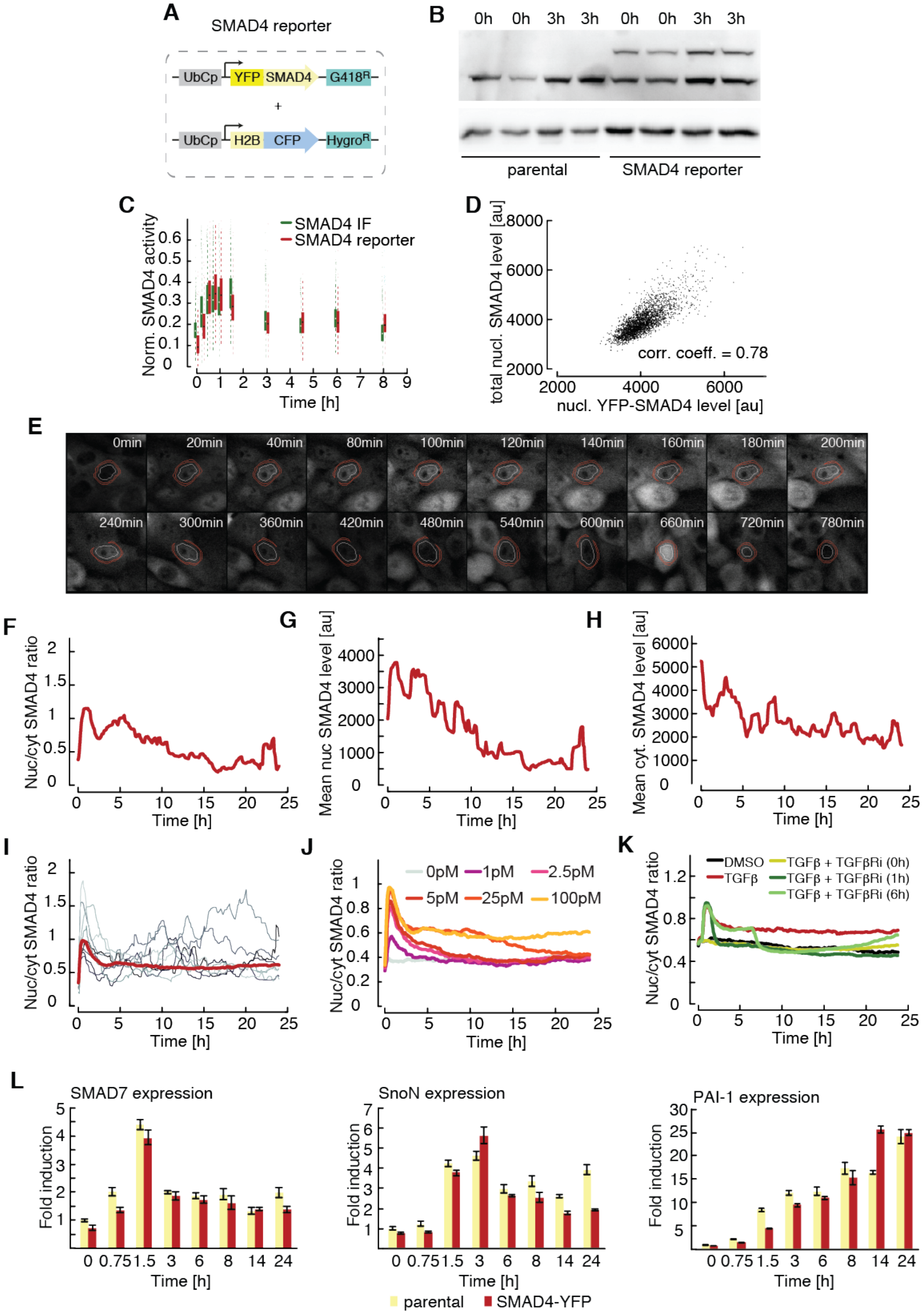

Appendix Figure S2. Dynamics and variability of SMAD4 signaling in single cells

(A) Fluorescent reporter system to measure SMAD signaling dynamics in individual

cells. SMAD4 was fused to the yellow fluorescent protein mVenus (YFP) under the control of the human Ubiquitin C promoter (UbCp) with the selection marker G418 (Geneticin). As a nuclear marker, histone 2B (H2B) was fused to the cyan fluorescent protein mCerulean (CFP) under the control of UbCp with the selection marker hygromycin.

(B) Western blot analysis of endogenous and YFP-tagged SMAD4 in a stable clonal reporter cell line and the corresponding parental cell line. Cells were stimulated with 100 pM TGFβ1 and analyzed after 3h. GAPDH was used as a loading control.

(C) Comparison of endogenous SMAD4 and SMAD4-YFP translocation. The nuc/cyt ratio of SMAD4-YFP upon 100pM TGFβ stimulation was measured in reporter cells by time-lapse microscopy at the indicated time points (green); the nuc/cyt ratio of endogenous SMAD4 was measured in parental MCF10A cells by immunofluorescence (IF, red) under the same conditions. Data was normalized by minimum subtraction and division through the overall maximum.

(D) Correlation between endogenously expressed SMAD4 and transgenic YFP-SMAD4. In the same individual SMAD4 reporter cells treated with 100pM TGFβ for 1.5h, nuclear endogenous SMAD4 was measured by immunofluorescence and compared to the nuclear fluorescence intensity from YFP-SMAD4. Both measures are highly correlated (Pearson's correlation, n=5500).

(E-H) Live-cell time-lapse microscopy images of MCF10A cells expressing YFP-SMAD4 following treatment with 100pM TGFβ (E). White framing indicates the segmented nucleus, the estimated cytoplasmic area is represented by red annuli. The indicated cell was tracked over 24h and the corresponding nuc/cyt ratio (F), nuclear (G) or cytoplasmic (H) fluorescence intensity of YFP-SMAD2 plotted over time.

(I) Time-resolved analysis of SMAD4 nuclear/cytoplasmic localization for eight individual cells (thin lines) compared to the median nuc/cyt SMAD4 ratio (thick line) upon stimulation with 100pM TGFβ. See Appendix Table S1 for number of cells analyzed.

(J) Median nuc/cyt SMAD2 ratio of cells stimulated with varying concentrations of TGFβ over 24h. See Appendix Table S1 for number of cells analyzed.

(K) Median nuclear-to-cytoplasmic SMAD4 ratio for reporter cells stimulated with 100pM TGFβ and treated with TGFβRI Kinase inhibitor (SB431542) at indicated time points. At all time points, SMAD4 nuclear translocation was dependent on TGFβ receptor activity. See Appendix Table S1 for number of cells analyzed.

(L) Expression of SMAD target genes in parental and SMAD4 reporter cell lines. Expression kinetics of the SMAD target genes SMAD7, SnoN and PAI-1 upon 100pM TGFβ stimulation were measured by qPCR in the indicated cell lines. β-Actin was used as an internal control. Error bars indicate standard deviation of technical triplicates.

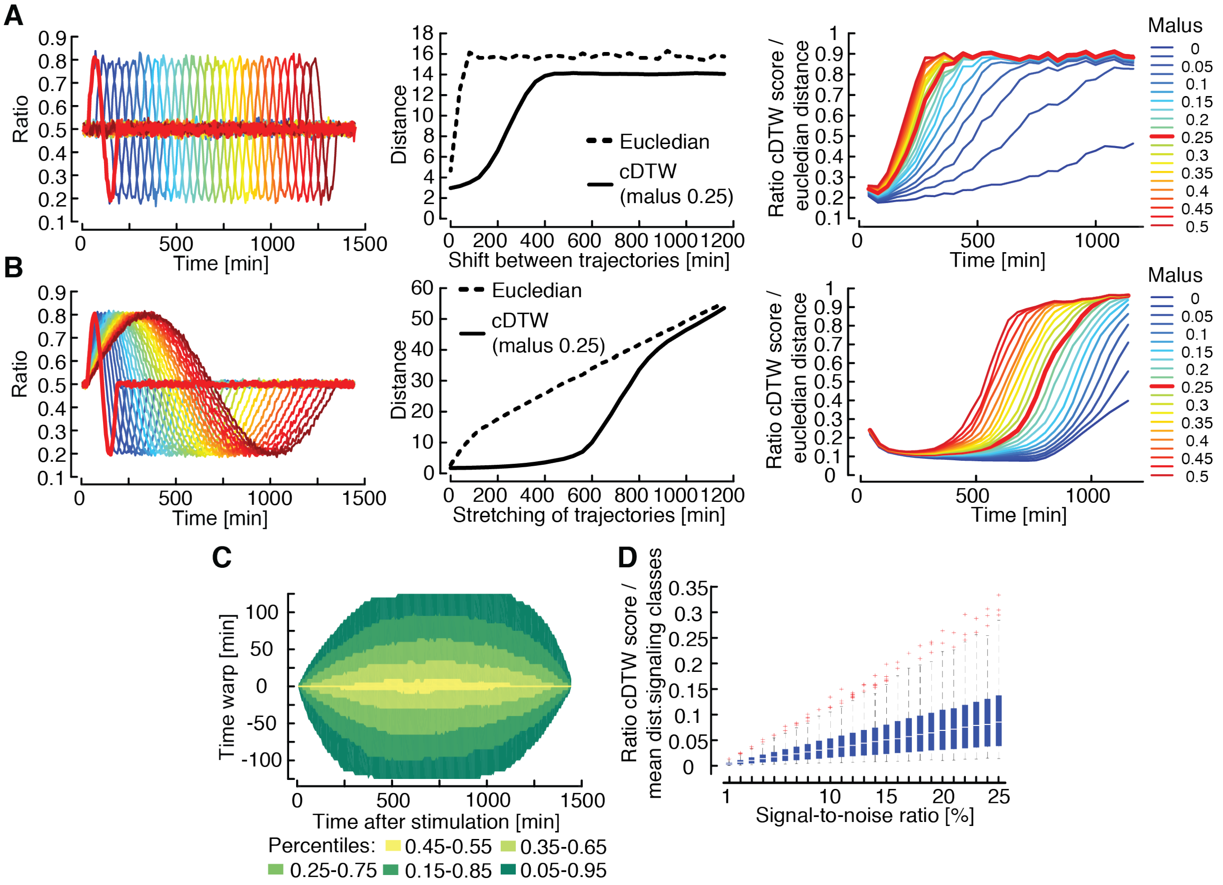

Appendix Figure S3. Performance and robustness of constrained dynamic time warping

(A) The parameter *malus* restricts to which extent cDTW compensates shifts in time. An artificial trajectory was systematically shifted in time (left panel) and resulting distances were measured using Euclidian distance (middle panel, dashed line) or cDTW (middle panel, *malus* = 0.25, solid line). With increasing shifts the cDTW score grows gradual until it reaches values similar to the Euclidean distance at around 400min. The parameter *malus* controls how cDTW scores approach Euclidian distances (right panel). Note that Euclidean distance stays constant as soon as the patterns do not overlap anymore.

(B) The parameter *malus* restricts to which extent cDTW compensates stretching in time. An artificial trajectory was systematically stretched in time (left panel) and resulting distances were measured using Euclidian distance (middle panel, dashed line) or cDTW (middle panel, *malus* = 0.25, solid line). After a lag phase, increased stretching leads to gradually increasing cDTW scores. However, cDTW scores only approach Euclidean distance when trajectories are stretched by about 800min. This time frame can be adjusted by the parameter *malus* (right panel). Note that Euclidean distance grows gradually with stretching in contrast to shifting (see above).

(C) Observed time shifts in SMAD2 trajectories. cDTW distances between SMAD2 trajectories treated with varying concentrations of TGFβ were calculated using a Sakoe-Chiba band which restricts alignments to a maximum of 250 minutes (compare Figure

EV2C). Alignments are mostly restricted to time points less than 75 minutes apart.

(D) cDTW is robust to experimental noise. Measured trajectories were distorted by adding white noise according to the indicated signal-to-noise ratio. cDTW distances between measured and distorted trajectories were calculated and normalized to the average distances between observed signaling classes. Even with a high signal-to-noise ratio of 25%, cDTW scores remained below 10% of the average distance between observed signaling classes.

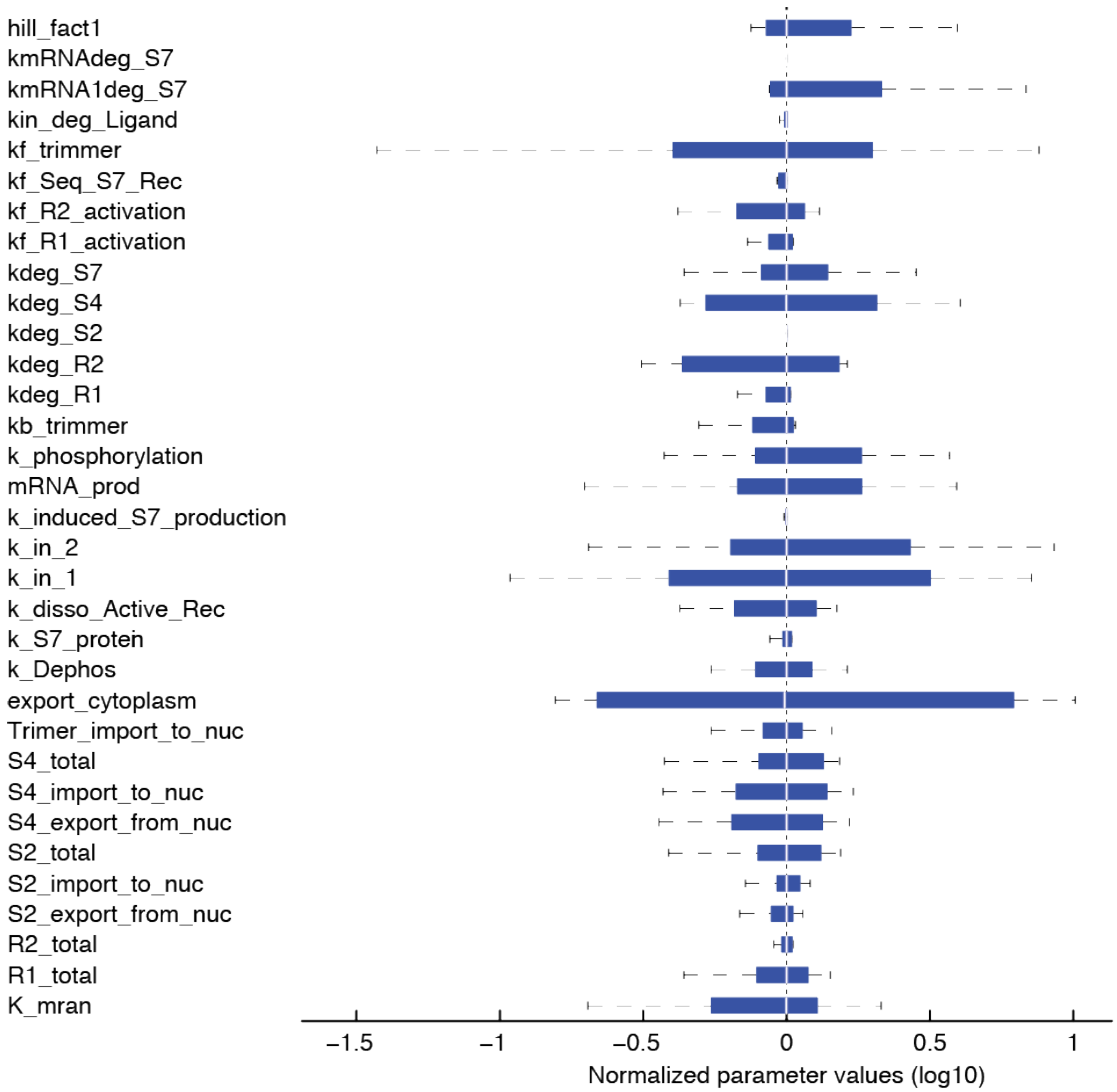

Appendix Figure S4. Parameter distributions from 30 independent population average model fits with similar goodness-of-fit

Distributions of biochemical parameters and protein concentration values from 30 independent fits to population average data with similar goodness-of-fit obtained from local multi-start optimization (see Appendix Table S3-S5, Table EV1). Each parameter is normalized to the median value from the 30 fits; distributions are shown in log10 scale (x-axis).

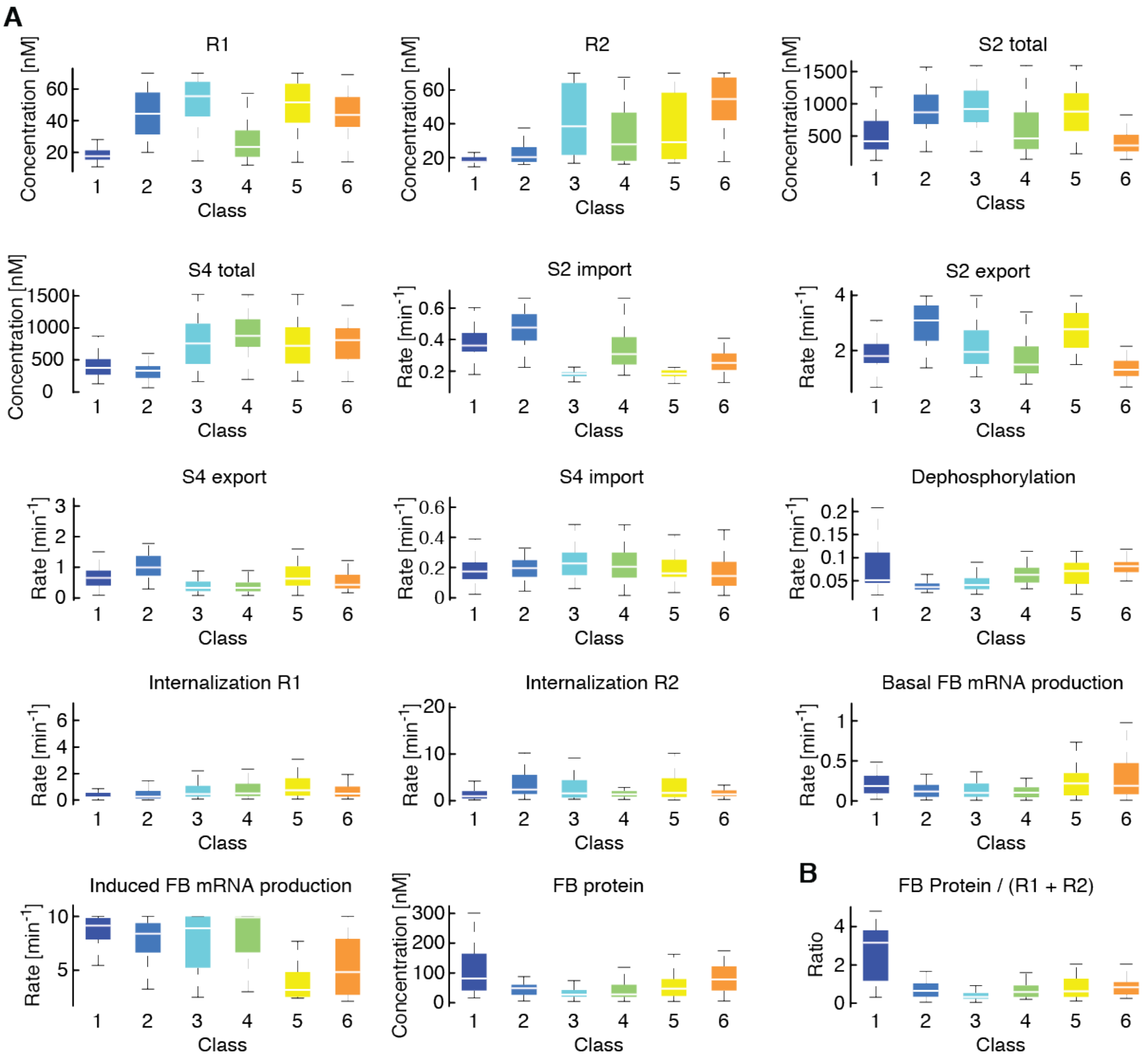

Appendix Figure S5. Distributions of parameter values for 30 independent fits to the signaling class dynamics with similar goodness-of-fit

(A) Distributions of model parameters allowed to vary between signaling classes among 30 independent fits with similar goodness-of-fit. The 30 fits to the six signaling classes were generated by individually fitting signaling classes as in Figure 5A from each of the 30 independent population-average fits (see Appendix Supplementary Methods).

(B) The ratio of feedback protein concentration to the sum of receptor concentrations distinguishes non-responding cells (Class 1) from responding cells (Class 2-6). The ratio was calculated from the values shown in A.

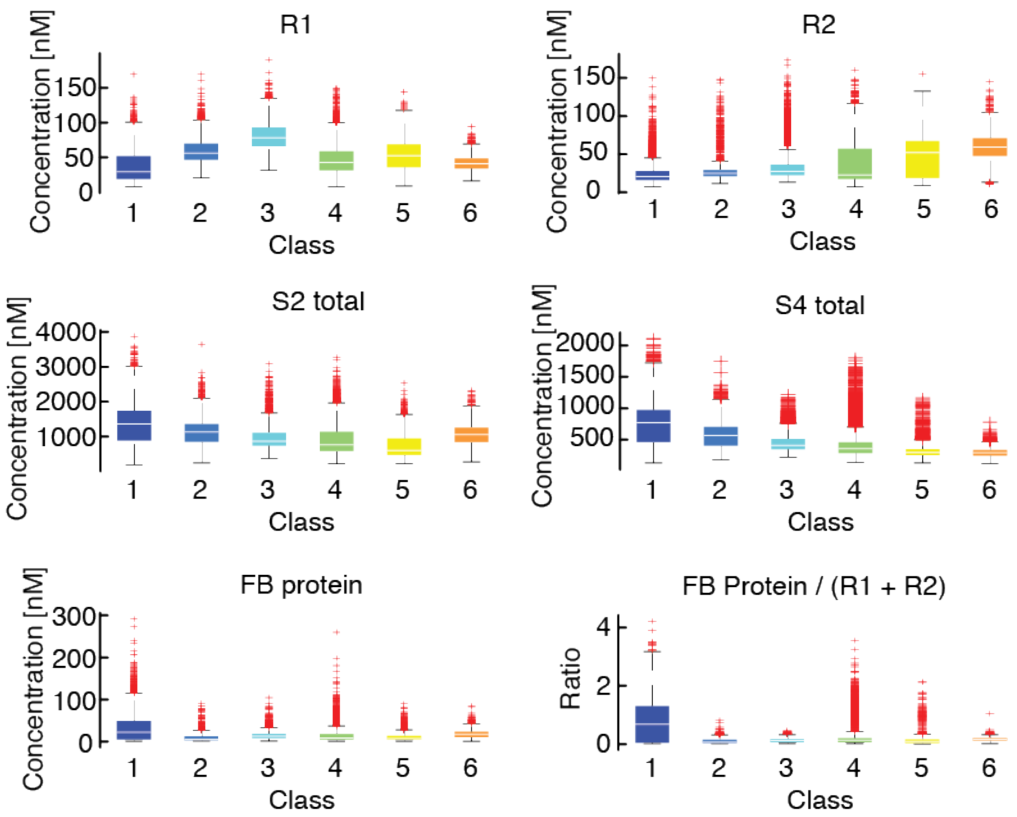

Appendix Figure S6. Distributions of signaling protein concentrations in an artificial cell population

An artificial cell population was assembled according to the observed proportion of signaling classes using calibrated noise levels (Figs. 4A and 5C-E). The response to a 5 pM TGFβ stimulus was simulated and trajectories mapped back to previously observed signaling classes. The distributions of selected signaling protein concentrations and for the feedback-to-receptor ratio are shown for artificial cells of each signaling class.

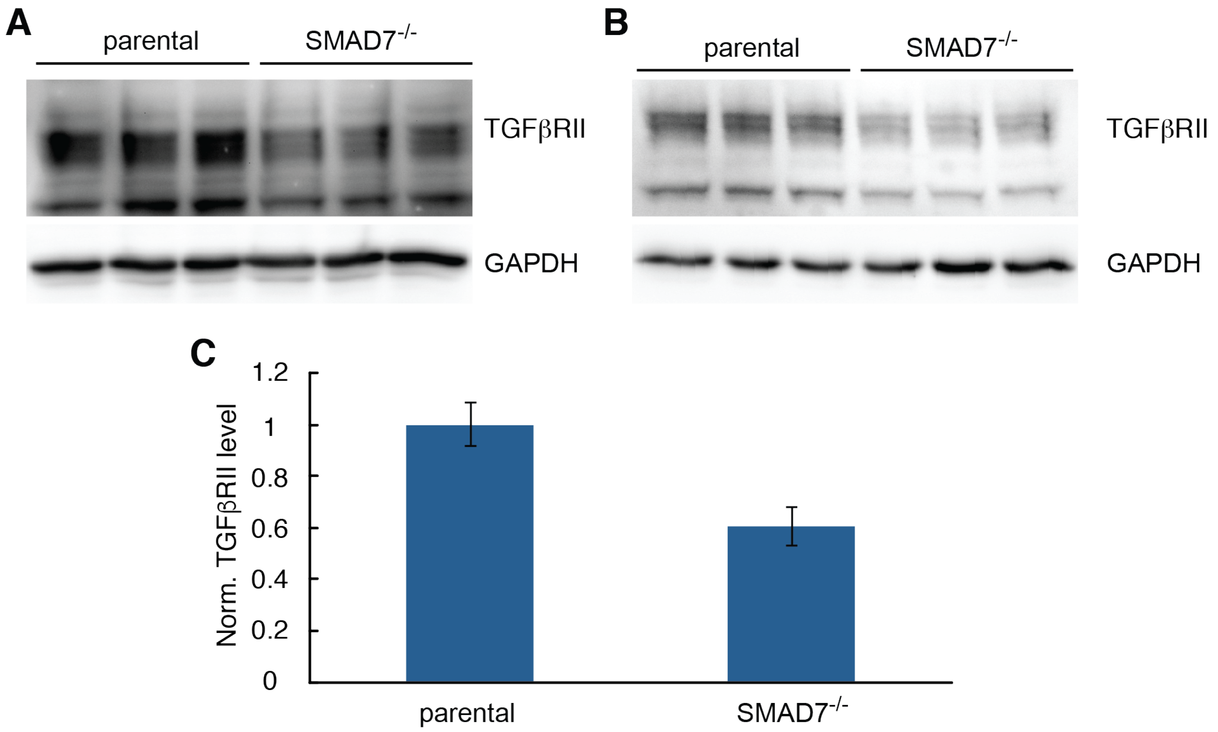

Appendix Figure S7. TGFβ receptor levels are decreased in SMAD7 knock-out cells

(A-B) Western blot analysis of TGFβRII levels in SMAD7 knock-out and parental cells. GAPDH was used as loading control. Each lane represents a biological replicate. (A) and (B) represent technical repeats.
(C) Quantification of western blot analysis shown in (A) and (B).

I. List of time-lapse microscopy experiments

Appendix Table S1: List of time-lapse microscopy experiments and the number of tracked cells per condition.

**SMAD2 translocation dynamics - TGFβ titration**	**control**	**TGFβ 1pM**	**TGFβ 2.5pM**	**TGFβ 5pM**	**TGFβ 25pM**	**TGFβ 100pM**
	358	395	314	295	351	352
**SMAD2 translocation dynamics - Receptor inhibition (100pM TGFβ)**	DMSO	TGFβ 100pM	TGFβ Inhibitor (30 min pre)+ 100pM TGFβ	TGFβ Inhibitor + 100pM TGFβ	100pM TGFβ + TGFβ Inhibitor (1h post)	100pM TGFβ + TGFβ Inhibitor (6h post)
	163	214	421	397	318	258
**Cell Cycle 48h, TGFβ at 24h**	control	TGFβ 1pM	TGFβ 2.5pM	TGFβ 5pM	TGFβ 25pM	TGFβ 100pM
	231	89	215	192	250	154
**SMAD4 translocation dynamics - TGFβ titration**	control	TGFβ 1pM	TGFβ 2.5pM	TGFβ 5pM	TGFβ 25pM	TGFβ 100pM
	196	191	213	311	236	275
**SMAD4 translocation dynamics - Receptor inhibition (100pM TGFβ)**	DMSO	100pM TGFβ	Inhibitor (30min pre)+ TGFβ	TGFβ + Inhibitor	TGFβ + Inhibitor (1h post)	TGFβ + Inhibitor (6h post)
	161	246	367	465	402	521
**SMAD2 G2 arrest R03306**	100pM TGFβ	100pM TGFβ + 3uM RO3306	1h pre 3uM RO3306 + 100pM TGFβ	2h pre 3uM RO3306 + 100pM TGFβ	4h pre 3uM RO3306 + 100pM TGFβ	8h pre 3uM RO3306 + 100pM TGFβ
	1132	1167	1704	1540	1327	837
**SMAD2 translocation dynamics - TGFβ titration replicate**	control	1pM TGFβ	2,5pM TGFβ	100pM TGFβ	25pM TGFβ	5pM TGFβ
	275	759	666	664	699	574
**SMAD 7 kd TGFβ Titration SMAD2**	control	1pM TGFβ	2,5pM TGFβ	100pM TGFβ	25pM TGFβ	5pM TGFβ
	487	680	833	654	784	623
**Cell Density – Cells per well**	25000	50000	75000	150000	300000	600000
	141	207	230	314	371	387
**100pM TGFβ + DRB**	DRB 0pM TGFβ -15min	DRB 100pM TGFβ -15min	100pM TGFβ	100pM TGFβ 30min post DRB	100pM TGFβ 45min post DRB	100pM TGFβ 1,5h post DRB
	880	1034	1224	1325	1363	1244
**5, 25 and 100pM TGFβ + DRB**	15min pre 100uM DRB + 2.5pM TGFβ	15min pre 100uM DRB + 25pM TGFβ	15min pre 100uM DRB + 100pM TGFβ	DMSO + 100pM TGFβ	DMSO + 25pM TGFβ	DMSO + 2.5pM TGFβ
	1250	1528	1167	1579	1425	1480
**SMAD2 translocation dynamics - Restimulation (100pM TGFβ)**	0h 100pM TGFβ	0h 3h 100pM TGFβ	0h 6h 100pM TGFβ	0h 3h 6h 100pM TGFβ	6h 100pM TGFβ	3h 100pM TGFβ
	244	277	269	237	264	278
**Restimulation 5pM TGFβ**	0h 100pM TGFβ	0h 3h 100pM TGFβ	0h 6h 100pM TGFβ	0h 8h 100pM TGFβ	3h 100pM TGFβ	8h 100pM TGFβ
	758	568	511	521	762	538

**DRB + 5pM TGFβ**	5pM TGFβ	5pM TGFβ + 3uM RO3306	1h pre 3uM RO3306 + 5pM TGFβ	2h pre 3uM RO3306 + 5pM TGFβ	4h pre 3uM RO3306 + 5pM TGFβ	8h pre 3uM RO3306 + 5pM TGFβ
	1132	1167	1704	1540	1327	837
**60 h Replicate 1**	control	TGFβ 1pM	TGFβ 2.5pM	TGFβ 5pM	TGFβ 25pM	TGFβ 100pM
	412	234	276	215	220	210
**60 h Replicate 3**	control	TGFβ 1pM	TGFβ 2.5pM	TGFβ 5pM	TGFβ 25pM	TGFβ 100pM
	396	602	407	461	587	282
**60 h Replicate 3**	control	TGFβ 1pM	TGFβ 2.5pM	TGFβ 5pM	TGFβ 25pM	TGFβ 100pM
	515	357	250	299	402	361
**SMAD2 translocation dynamics - Restimulation (2.5pM TGFβ)**	0h 2.5pM TGFβ	0h 3h 2.5pM TGFβ	0h 6h 2.5pM TGFβ	6h 2.5pM TGFβ	3h 2.5pM TGFβ	1.5h 2.5pM TGFβ
	222	241	181	237	261	335
	TGFβ 100pM					
**sister cells replicate 1**	989					
**sister cells replicate 2**	938					
**sister cells replicate 3**	1550					
**sister cells replicate 4**	1440					
**sister cells replicate 5**	1417					
**sister cells replicate 6**	796					
**sister cells replicate 7**	647					
**sister cells replicate 8**	1208					
**sister cells replicate 9**	2050					
**sister cells replicate 10**	1115					
**sister cells replicate 11**	745					

II. Single cell data analysis

II.A Image analysis and cell tracking

Cells were tracked throughout an image series using custom-written Matlab (MathWorks) scripts based on code developed by the Alon lab (Cohen *et al*, 2008) and the CellProfiler project (Carpenter *et al*, 2006). We first applied flat field correction and background subtraction to raw images before segmenting individual nuclei from nuclear marker images using automated thresholding with Otsu's method and seeded watershed algorithms. Segmented cells were then initially assigned to corresponding cells in following images using a greedy match algorithm. The resulting tracks were computationally examined for inconsistencies such as merging or splitting of nuclei. When inconsistencies were detected, segmentation was optimized with altered parameters and variations of watershed algorithms. After optimization, we repeated cell tracking using the same greedy match algorithm. Only cells tracked from the first to last time point were considered. For most analyzes, we tracked cells in forward direction from the first to the last time point. Upon division, we followed the daughter cell closest to the last position of the mother and merged tracks from mother and offspring. For sister cell analyzes, cells were tracked backwards from the last to the first time point, tracks from offsprings and mothers were again merged. As a consequence, tracks of sister cells are identical before cell division. We quantified nuclear fluorescence intensity and measured the fluorescence intensity in the cytoplasm using a 4-pixel wide annulus around the nucleus. The annulus was grown from nuclear segmentations avoiding overlap with neighboring cytoplasmic and nuclear regions. Due to a lack of consistent cytoplasmic markers, we could however not exclude that it contains extracellular regions. To minimize the influence of an incomplete cytoplasmic segmentation, we determined the nuclear to cytoplasmic SMAD2 ratio using the median fluorescence intensity of the estimated cytoplasmic region for each cell over time, which is more robust against fluctuations than the mean intensity. We compared this measure to corresponding ratios calculated using the first or third quartile (Appendix Figure S1A). While absolute values varied as expected, all measures were linearly correlated with correlation coefficients above 0.95, indicating that ratios are robust against variations in cytoplasmic fluorescence intensities. We further analyzed whether the size of the cytoplasmic region influenced nuc/cyt SMAD ratios by measuring how they change with a decreased width of the annulus (Appendix Figure S1B). Deviations were minimal for

untreated cells and increased only slightly at higher stimulus levels due to the larger dynamic range of measurements. We globally analysed the normalized deviation of nuc/cyt SMAD ratios for calculations with increased and decreased width of the cytoplasmic annulus and observed changes of less than 1% (Appendix Figure S1C).

II.B Data preprocessing

Before analysis, we preprocessed our data with custom made filters to remove technical noise and signals from biological events unrelated to the induced response. To remove spikes and sustained intensity shifts caused by segmentation and tracking errors, we identified those features based on cell specific thresholds defined by the standard deviation of the processed trajectory and replaced them by linear interpolation. Spikes and sustained shifts are defined either by a decrease followed by an increase or an increase followed by a decrease both greater than the standard deviation of the processed trajectory. Removal of Spikes and sustained shifts is done iteratively. This led to an increased signal-to-noise ratio due to local smoothing, emphasizing slower changing biological responses. As the nuclear envelop breaks down during mitosis and morphological changes lead to increased auto-fluorescence, we disregarded signals measured during cell division and removed them from the trajectories by interpolation. To identify cell divisions, we normalized for each cell the nuclear area and the integrated fluorescence intensity of the nuclear marker to their respective means, smoothed them by the approach described above and combined the two trajectories by averaging. We then applied a 1D Prewitt filter (Lipkin, 1970) (length 75 minutes) to the combined trajectory to amplify signal discontinuities that correspond to cell divisions. Cell divisions events corresponded to a value of the Prewitt filtered trajectory exceeding a manually selected threshold based on several hundred visually detected cell divisions.

II.C Measuring cell-to-cell dissimilarity using Dynamic Time Warping

To compare single cell trajectories a measure for similarity/dissimilarity must be established. Because of the known weakness of the Euclidean distance especially the sensitivity to distortion in time axis, we use Dynamic Time Warping (DTW), a standard method to address the temporal alignment (Sakoe & Chiba, 1978; Berndt & Clifford, 1994; Keogh & Pazzani, 2000) that overcomes the limitations of the Euclidean distance (Figure EV2C) . DTW results in a non-linear mapping of one trajectory to another by warping the time axis iteratively until an optimal match between the two trajectories

with respect to the used metric is found (Figure EV2C). DTW is based on dynamic programming, which allows for solving complex problems by breaking them down into simpler sub-problems. DTW takes into account that distinct patterns within trajectories may differ in frequency and length and affect the shape of an individual time courses. Application of DTW adjusts positions of these specific patterns and local differences between two trajectories and aligns them in a non-linear manner. This is a great advance compared to simpler methods like measuring Euclidean distances, which do not take temporal heterogeneity into account (Figure EV2C). In the standard DTW implementation, only the length of the trajectories limits the temporal alignment. However, from a biological perspective it is only useful to map features of two-time series onto each other if they are within a certain time window. Therefore, we added a stiffness constrain to the elastic measure along the time axis using increasing penalties for longer stretches. To this end, we tracked the distance of the temporal alignment by an additional matrix within the recurrence of our DTW algorithm. We will refer to our approach as constrained DTW (cDTW). How strong the stretching gets penalized can be controlled by the parameter *malus*. Setting *malus* to zero corresponds to an unconstrained DTW. With increasing *malus* the results are driven in the direction of the Euclidean distance. To analyze the performance of cDTW, we generated an artificial noisy time series, systematically altered it by shifting or stretching and compared resulting cDTW scores with varying strength of the parameter *malus* to the Euclidian distance (Appendix Figure S3A-B). This analysis indicated that without constrain (*malus* = 0), even temporally distant or highly distorted features were aligned, leading to low cDTW scores. Increasing *malus* restricted the ability of the algorithm to align features. When using a *malus* of 0.25, features shifted by about 300min were no longer recognized as similar and cDTW scores approached Euclidian distance. Similar effects were observed for stretched features, although on slightly longer time scales. However, even if distant features were aligned, cDTW scores increased with increasing shift / stretch. In our analysis, we chose a value of 0.25 for *malus* as this constrained cDTW on the time scales of the first SMAD response to TGFβ stimulation (compare Figure 1E-F).

We then used cDTW to generate a dissimilarity matrix (Figure EV2D) by computing pairwise dissimilarities between single cells. As computing pairwise cDTW scores for thousands of trajectories is a very computationally intensive task, we restricted calculations to a Sakoe-Chiba band (Sakoe & Chiba, 1978) and used parallel computation. The Sakoe-Chiba band is used to speed up the computation by constraining

the warping path. The parameter *bandWidth* controls how far the recurrence can deviate from the main diagonal. The smaller the band the bigger the speed up effect apart from that the less flexible the alignment. Employing a Sakoe-Chiba band onto the titration dataset further bounded temporal alignments at 125 min. As a result, we observed a "time warp" of less than 75min (mean: 46min) for most data points in the pairwise comparison (Appendix Figure S3C).

To analyze the robustness of the cDTW measure to experimental noise, we added defined quantities of white noise to measured single cell trajectories and calculated cDTW distances to the unaltered time series. We then normalized the resulting cDTW distances between noisy and unaltered trajectories to the average distances between the experimentally observed signaling classes (Figure S3D). From this analysis, we conclude that experimental noise will have only a minor effect on cDTW-based clustering, as even high signal-to-noise ratios of up to 25% led to cDTW distances between noisy and unaltered trajectories that correspond to only around 10% of the mean distances between the observed signaling classes.

```
cDTW(t1:array[1...n],t2:array[1...n], malus:double, bandWidth:int)
    //init
    dpMatrix      = array[0...n, 0...n]
    dpMatrix[1:n,0] = Inf
    dpMatrix[0,1:n] = Inf
    dpMatrix[0,0]   = 0
    sMatrix       = array[0...n, 0...n]
    sMatrix[0:n,0] = Inf
    sMatrix[0,1:n] = Inf
    sC = quantile(dist(t1,t2),malus)
    //Recurrence
    for i = 0:n-1
        for j = max(i-bandWidth,0):min(i+bandwidth,n-1)
            cost = dist(t1[i],t2[j])
            [value idx] = min(dpMatrix[i,j+1] – min(-sC,sC*sMatrix[i,j+1]),
            dpMatrix[i+1,j] + max( sC,sC*sMatrix[i+1,j]),
             dpMatrix[i-1,j-1] )
            dpMatrix[i,j] = cost+value
            if(idx == 0)
                 sMatrix[i,j] = min(sMatrix[i-1,j]-1,-1)
            elseif(idx == 1)
                sMatrix[i,j] = max(sMatrix[i-1,j]+1,1)
            else
                sMatrix[i,j] = 0;
            end
        end
    end
  return dpMatrix[n,n]
```

```
end
```

Pseudo-Code for cDTW calculation

II.D Clustering of single cell trajectories

Based on the pairwise dissimilarity matrix estimated by cDTW we applied clustering to group cells with similar dynamic patterns. We used a two-stage clustering approach composed of a primary hierarchical clustering step followed by a centroid model based refinement step. For hierarchical clustering we employed the agglomerative Ward's method (Ward, 1963) (Appendix Figure S3E). Ward's method minimizes the within-cluster variance based on the sum of squares between two clusters. We used the output of Ward's method to extract the linkage of the objects and assign, based on this, each trajectory to clusters. Ward's method yields very homogeneous clusters and is the most powerful agglomerative method. The initial clustering was refined with a centroid model based algorithm utilizing the Hausdorff distance (Groß, 1915): iteratively, we computed the Hausdorff distance of each trajectory to the clusters and assigned the trajectory to the closest cluster until we reached optimal assignments or the maximal number of iterations. We introduced the second clustering step as a sanity check based on the idea that the number of iterations is low if the hierarchical clustering result classified the data well. For our data, a single iteration was sufficient, after which more than 95% of the trajectories were still assigned to their initial cluster.

Good clustering represents a useful generalization of the data that emphasizes inherent features within clusters to substantiate a hypothesis. Choosing the correct number of clusters is an important but intricate task lacking a ground truth. Nevertheless, several techniques for choosing a reasonable cluster number and measuring object allocation quality have been proposed. All these validation techniques suffer from specific limitations due to the complex interaction of clustering algorithms, validation measures and underlying data and should therefore be used mainly as guidance (Handl *et al*, 2005).

We used the *jump* method (Sugar & James, 2003) for choosing cluster number and the *silhouette* method (Rousseeuw, 1987) for visualizing clustering quality (Figs. 2E, EV2F and EV5B). The *jump* method uses distortion, a quantity that measures the average distance between each observation and its closest cluster center. The distortion value depends on the number of clusters d_k. The largest jump defined as the difference between the distortion for k and k+1 clusters is an indicator for the number of clusters

that exist in the underlying dataset. The *jump* method indicated that choosing a clustering with three or six signaling classes would be a good choice for our data (Figure EV2F). Because three clusters would not provide us with the resolution we needed we decided to employ six clusters throughout this study. From our perspective six clusters were representing best the dynamical heterogeneity we aimed to analyze.

The *silhouette* method quantifies how similar an object is to objects within its own cluster compared to objects from other clusters and gives a useful graphical representation to assess allocation quality. This is achieved by measuring the intracluster divergence (tightness) compared to intercluster divergence (seperation). The silhouette coefficient can range from -1 to 1. The higher the silhouette coefficient for all objects the better the clusters are separated. The silhouette method can be applied to many algorithms for partitioning data into clusters. In Figure 2E we compared our assignment to the six signaling classes with the allocation to the different conditions (extracellular ligand concentration). The assignment of single cell trajectories to signaling classes provides a different separation than grouping cells according to ligand dose.

II.E Local cell density

An environmental parameter that may influence the cellular response to TGFβ is local cell density. Local cell density is a weighted measure of how many neighboring cells each cell has over time and how close these neighbors are. To quantify the density, we take all segmented nuclei from the nuclear marker images into account. For each tracked cell we compute the local density as the distance-dependent weighted sum of all neighboring cell within a radius of 200px (Appendix Figure S4E right). We use a sinusoidal weighting function to ensure that closer cells have a higher weight than cells further away (Appendix Figure S4E left). If distance between two cells is lower than 200 px the density score is given by $0.5(\cos(\text{distance between cells}/200px\ \pi)+1)$, else the density score is 0. We discard cells within 200px of the outer image boundaries of each frame before the analysis to avoid underestimating local cell density.

To quantify how cell cycle and local cell density contribute to heterogeneity in cell responses we used the concept of mutual information (Figs. S4G and S4H). Mutual information is a measure of information that one random variable contains about another random variable. In a first step we applied non-uniform binning into B bins, such that each bin is equally filled. Binning was done separately for the

nucleocytoplasmic ratio and the cell density estimates. Based on this binning we estimated the marginal probability distributions $\{p_1^{ratio}, \dots, p_t^{ratio}\}$ and $\{p_1^{density}, \dots, p_t^{density}\}$ for each time point *T = {1,...,t}* and the joint probability distributions $\{p_{1,1}^{ratio,density}, \dots, p_{t,t}^{ratio,density}\}$ for each pair of time points T x T. The Mutual Information *MI* of the two discrete variables was computed for all combination of time points by applying $MI(i,j) = \sum_{ratio \in B} \sum_{density \in B} p_i^{ratio} \log\left(\frac{p_{i,j}^{ratio,density}}{p_i^{ratio} p_j^{density}}\right)$. To provide an intuitive interpretation of MI, we scaled the result by the sum of the two entropies, $MI(i,j)/(H(p_i^{ratio})+H(p_j^{density}))$, on the basis of *MI(X;Y) = H(X)+H(Y)-H(X,Y)*. This indicates the fraction of the observed heterogeneity that can be explained by cell cycle (Fig S4G) or cell density (Fig S4H).

II.F Motility

Locomotion can be summarized by the distribution of speed and direction change. The distance each cell has moved was calculated with respect to the direction change (Appendix Figure S3G and S3H). We get the angle from the actual, previous and next position of a cell. We have computed effect sizes and the confidence intervals of median fold change of the directed motility (-30° to 30°) using permutation testing (Appendix Figure S3I and S3J). For plotting we have binned the results into bins of 20°.

II.G Sister cell analysis

One potential source of cell-to-cell variability are differences in the cellular state. Sister cell analysis can shed a light on this issue due to the assumption that the state of recently divided cells is similar. Sister cell analysis (Figure 3E) was performed on cells stimulated with 100pM TGFβ and imaged for 48h. To increase the statistical power of the analysis, we combined data from 11 experimental replicates. In total, we used an initial population of about 6000 cells, giving rise to 13.000 cells after 48h as the result of about 7000 recorded cell division events. To investigate how sister cells differ from cells with distinct progenitors we again relied on cDTW. We calculated the similarity of two SMAD2 nucleo-cytoplasmic ratio trajectories over time, using a window of length 4h (this are 48 time points at the used sampling rate of 5 min) and generated a series of similarity measures *s*, by moving the sliding window along the time axis starting from time of division *i* (*s[i] = dtw(t1[i:i+48],t2[i:i+48]*). We generated a second data set using 'fake

sister cell' cells, that coincidentally shared the same SMAD2 nucleo-cytoplasmic ratio at a time point ('fake divisions'). To estimate if sister cells are more similar over time than the set of coincidentally identical 'fake sister cells', we used cDTW similarity measure as described above to compare the sister cells and the 'fake sister cells' with randomly selected cells from the non-sister subpopulation.

By using a bootstrapping testing procedure, we compared the different cell population and estimate the size of the observed effect (difference in average cDTW score) and corresponding 95% confidence intervals. This was done by comparing 100 randomly picked measures from each population and repeating this 1000 times. We found the SMAD2 response to be more correlated in sister cells than in 'fake sisters' or randomly selected cells over a longer period of time.

II.H Mapping single cell trajectories to signaling classes

In the course of our work it was necessary to map several datasets onto the six clusters we had originally derived from the wildtype titration dataset (Fig 2C). To this end we used a heuristic approach: The mapping is done by computing pairwise Euclidean distances between all single cell trajectories from the original TGFβ titration data set and all single-cell trajectories from other datasets e.g. data shown in Figure 3B, the mathematical model or the KD-cell line. Each single-cell trajectory from these datasets was assigned to the cluster to which the closest single-cell trajectory from the original dataset belongs.

II.I TGFβ measurement

In Figure 4D, we estimated the amount of TGFβ in the cell culture medium at different time points post stimulation to validate our model prediction that cell-mediated TGFβ degradation induces signal adaptation. We used Mink lung epithelial cells (MLECs) stably transfected with a reporter containing a truncated PAI-1 promoter (3TP promoter with three consecutive TPA response elements) fused to the firefly luciferase gene and cultured them in 96 well plates using DMEM (Abe *et al*, 1994). Supernatants from live-cell microscopy experiments were removed at defined time points and added in triplicates to MLEC reporter cells. After incubation overnight, cells were lysed and thawed. Luciferase activity was measured by 10-second well readings on a 96 well format luminometer. To create a standard curve, we incubated reporter cells with known TGFβ concentrations: triplicate measurements were performed for 8 different TGFβ

concentrations (0, 10, 20 40, 60, 80, 100 and 200 pM) in RPMI without phenol red including insulin, EGF, cholera toxin, HEPES, P/S, Glutamax and horse serum. To increase robustness we used permutations of the measures to generate several hundred independent fits to a modified Hill model $f(x) = k\frac{x^b}{x^b+a^b} + c$. The Hill equation was chosen to also reflect nonlinear parts of the calibration curve.

To measure TGFβ depletion, MCF10A cells were seeded two days before stimulation with 25 pM or 100 pM and supernatant was collected 1, 2, 3, 4, 5, 6, 7, 8, 9, 10, 13, 14, 16, 18 and 24 h post stimulation and added to MLEC medium. The fit to the standard curve was used to convert measured relative luciferase activities into absolute TGFβ concentrations. The estimated TGFβ concentrations at different time points post stimulation were assembled into time courses and fitted using an exponential decay model $f(x) = ae^{bt}$ to estimate the TGFβ decay constant b. The estimated decay constant depended on the initial TGFβ concentration: for an initial concentration of 100pM, we observed a TGFβ half-life of $9(\pm 2)$h, for 25 pM initial concentration a half-life of $6(\pm 1)$h. This may be caused by biological effects such as non-linearity in TGFβ degradation or technical limitations such as saturation of the assay.

III. Population-average model of the SMAD signaling pathway

III.A. Model topology

We implemented a mathematical model of the SMAD signaling pathway to quantitatively understand SMAD2-YFP time-lapse imaging data obtained at different strengths of TGFβ stimulation. The model structure was derived based on literature knowledge (Schmierer *et al*, 2008; Zi *et al*, 2011; Vizán *et al*, 2013), and captures the information flow from the cell membrane to the nucleus as well as the transcriptional induction of feedback regulators. In the following, we will initially describe the biological assumptions underlying our model, and then provide detailed information about model calibration and how we quantitatively describe the cell-to-cell variability in the signaling network.

The TGFβ /SMAD signaling pathway model is schematically depicted in Figure 4B, and can be considered to consist of three modules: (i) the receptor trafficking model which comprises the ligand-induced activation and trafficking of transmembrane TGFβ receptors; (ii) the SMAD cycle which involves receptor-mediated phosphorylation of SMAD transcription factors and their shuttling between nuclear and cytoplasmic compartments; (iii) the transcriptional feedback module in which a generic negative feedback regulator (termed FB in Figure 4B) is transcriptionally induced by nuclear SMAD complexes, and then inhibits TGFβ receptors. In the following, each of these modules will be described in detail. All model species and parameters are listed in Appendix Table S2 and S3, respectively.

Receptor trafficking module: TGFβ/SMAD signaling is initiated by binding of extracellular TGFβ (abbreviated as L in main Figure 4B) to its cognate transmembrane receptor, the TGFβ receptor 2 (R2) (López-Casillas *et al*, 1993; Boesen *et al*, 2002). We modeled this binding step as a reversible bi-molecular reaction (L + R2 <=> R2-L). The resulting receptor-ligand complex recruits the TGFβ receptor 1 (R1 + R2-L <=> R1R2-L) and internalizes in the endosome (R1R2-L => R1R2-Le) (Di Guglielmo *et al*, 2003) Receptor-ligand complexes are subject to autophosphorylation, thereby becoming active serine/threonine kinases to phosphorylate the downstream SMAD2(S2) transcription factors. Based on literature evidence, we assume in the model that only endosomal receptor complexes are enzymatically active and do not explicitly describe the autophosphorylation step which we assume to be fast (Zi *et al*, 2011). The activity of

endosomal receptor complexes can be terminated by irreversible dissociation of the ligand due to acidification in the endosomal compartment (R1R2-Le => R1e + R2e + Le). The endosomal ligand is then degraded in the lysosome (Le => Ø) (Mitchell *et al*, 2004), and this process occurs with linear kinetics (as all degradation reactions described further below). In contrast, the free endosomal receptors (R1e, R2e) can be recycled back to the plasma membrane. This cycle of receptor internalization and externalization (R1 <=> R1e; R2 <=> R2e) also operates in the absence of stimulation (Mitchell *et al*, 2004; Le Roy & Wrana, 2005; Razani *et al*, 2001).

Several endosomal receptor species are subject to degradation in the model (R1e => Ø; R2e => Ø; R1R2-L => Ø; R1R2-Le => Ø), as lysosomal degradation has been described for TGFβ receptors (Mitchell *et al*, 2004). For the case of cell-surface R1R2-L, we lumped raft-mediated endocytosis and subsequent lysosomal degradation into a single step in the model, and assumed that this degradation pathway can be enhanced by the generic negative feedback regulator (FB, see below) as described in the literature (Di Guglielmo *et al*, 2003). To balance the loss of receptors due to degradation, we included a constant influx of receptors to model the *de novo* synthesis of receptors (=> R1, => R2), reflecting synthesis of the transmembrane receptors via the secretory pathway (Zi *et al*, 2011).

SMAD activation and shuttling: In the absence of stimulation, SMAD2 (S2) is predominantly unphosphorylated and localized to the cytoplasm. Signaling is initiated by phosphorylation of S2 which is carried out by the endosomal receptor complex (R1R2-Le; see above). Phosphorylated S2 (pS2) then undergoes homotrimerization (three pS2 molecules) (Wu *et al*, 2001) or heterotrimerization (Inman & Hill, 2002) (two pS2 molecules and one SMAD4 (S4) molecule). *In vitro* studies showed that SMAD dimer intermediates are present only at negligible concentrations. Hence, SMAD homo- and heterotrimerization in the cytoplasm and nucleus were modeled as reversible, single step reactions (2 pS2 + S4 <=> $(pS2)_2S4$ and 3 pS2 <=> $(pS2)_3$) (Wegner *et al*, 2012; Chacko *et al*, 2004). Based on *in vitro* and *in vivo* studies (Chacko *et al*, 2004; Chen & Murphy, 2005; Schmierer & Hill, 2007), we assumed in the model that heterotrimerization occurs with higher affinity compared to homotrimerization.

S2 and S4 continuously shuttle between nucleus and cytoplasm even in the absence of stimulation (Schmierer *et al*, 2008). We therefore implemented cycles between nuclear and cytosolic SMAD pools (nS2 <=> S2, S4n <=> S4). TGFβ stimulation induces nuclear accumulation of S2 by reducing the nuclear export rate, while leaving the nuclear import

rate unaffected (Schmierer *et al*, 2008). The model was therefore implemented such that unphosphorylated and phosphorylated S2, as well as SMAD homo- and heterotrimer all possess equal nuclear import rates (S2 --> S2n; pS2 --> pS2n; $(pS2)_2S4$ --> $(pS2n)_2S4n$ and $(pS2)_3$ --> $(pS2n)_3$). We included a distinct rate constant for the import of monomeric S4 (S4 --> S4n), as this step was shown to be mechanistically different from import of S2 and SMAD trimers (Schmierer & Hill, 2005). SMAD trimers are exported less efficiently from the nucleus than monomeric unphosphorylated S2, because SMAD trimers are retained in the nucleus by binding to DNA and other transcription factors (Inman & Hill, 2002; Schmierer *et al*, 2008). Hence, we neglect the direct nuclear export of SMAD trimers in the model, and assume that they have to first decay into monomers before they are shuttled out of the nucleus. Note that some dissociation and dephosphorylation reactions of the SMAD trimers are not explicitly shown in the scheme in Figure 4B (see Appendix Table S2). Recent studies show dephosphorylation of an SSXS motif located on the binding interface of the heterotrimer ($(pS2n)_2S4n$) destabilizes the SMAD complex (Nishi *et al*, 2011), thus we assumed breakdown of $(pS2n)_2S4n$ through both unbinding (Schmierer *et al*, 2008) and dephosphorylation (Wegner *et al*, 2012) reactions.

TGFβ stimulation does not appear to strongly modulate the total S2 and S4 concentrations in MCF10A cells, as we observed stable levels of these proteins using Western Blotting (not shown). In our model, we assumed that they are produced with a constant rate throughout the time of our experiments (=> S2; => S4), and considered that all forms (e.g., phosphorylated vs. unphosphorylated or monomeric vs. complexes) can be degraded with the same rate (see Appendix Table S3). The balance between synthesis and degradation in the unstimulated state is ensured by calculating a steady state (see Section III.C).

Transcriptional feedback. TGFβ signaling is known to be controlled by transcriptional negative feedback loops (Legewie *et al*, 2008; Kavsak *et al*, 2000), and using the general transcription inhibitor DRB, we could show that these negative feedbacks operate in TGFβ-stimulated MCF10A cells as well (Figure 4E). In the model, feedback is implemented by assuming that nuclear SMAD heterotrimers induce the expression of a generic feedback regulator (FB) (note that these reactions are not shown in complete detail in Figure 4B, cf. Appendix Table S2): Expression of FB mRNA (=> FBmRNA1) occurs at a basal rate and can be enhanced by $(pS2n)_2S4n$. The nuclear FB mRNA is

degraded (FBmRNA1 =>) or exported into the cytoplasm (FBmRNA1 => FBmRNA). Cytosolic FB mRNA is degraded (FBmRNA =>) or translated into FB protein (=> FB). FB protein inhibits the signaling by disrupting the active receptor complex on the plasma membrane (R1R2-L) (Dijke *et al*, 1997; Kavsak *et al*, 2000). We modeled the action of the feedback regulator as a two-step process where at the first step FB and R1R2-L Rec forms an inactive complex (R1R2-L + FB => Inactive_Rec) which can no longer phosphorylate SMAD2 (Dijke *et al*, 1997). The Inactive_Rec breaks down either by dissociation (Inactive_Rec => R1R2-L + FB) or by degradation of its components (Inactive_Rec => R1 + R2 + FB; Inactive_Rec => R1 + TGFβ_in + FB; Inactive_Rec => R2 + TGFβ_in + FB).

III.B Model equations

The model simulations were performed using the following ordinary differential equations (ODEs).

d(x1)/dt = p1 + x8*p8 - x1*p14 - x7*x1*p7 + x10*p20*p9 + x10*p19*p11 + x3*p16*p14 .

d(x2)/dt = p2 - x2*p15 + x7*p6 + x10*p19*p11 + x4*p17*p15 - x5*x2*p5 .

d(x3)/dt = x9*p21 + x9*p9 + x9*p11 + x1*p14 - x3*p10 - x3*p16*p14 .

d(x4)/dt = x9*p21 + x9*p10 + x9*p11 + x2*p15 - x4*p9 - x4*p17*p15 .

d(x5)/dt = p47 + (x7*p6)/p48 - (x5*x2*p5)/p48 .

d(x6)/dt = x9*p21 + x9*p10 + x9*p9 - x6*p11 + x10*p20*p9 .

d(x7)/dt = x8*p8 - x7*p6 - x7*x1*p7 + x5*x2*p5 .

d(x8)/dt = x10*p13 - x8*p8 - x8*x18*p12 + x7*x1*p7 - x8*p18*p14 .

d(x9)/dt = x8*p18*p14 - x9*p10 - x9*p9 - x9*p11 - x9*p21 .

d(x10)/dt = x8*x18*p12 - x10*p13 - x10*p20*p9 - x10*p19*p11 .

d(x11)/dt = (p28*x19)/2 - p27*x11 + 2*p25*x14 - p43*x11 + p43*x14 + 2*p44*x14 + 2*p43*x15 - 3*p26*p24*x11^3 + 3*p49*p25*x15 - 2*x13*p24*x11^2 - x9*x12*p22*(p36*p23 - 1) .

d(x12)/dt = p3 - x12*p27 + (x22*p28)/2 - x12*p43 + x9*x12*p22*(p36*p23 - 1) .

d(x13)/dt = - x13*p24*x11^2 + p4 - x13*p29 + (x23*p30)/2 - x13*p44 + p25*x14 + p43*x14 .

d(x14)/dt = x13*p24*x11^2 - p31*x14 - p25*x14 - p43*x14 - p44*x14 .

d(x15)/dt = p26*p24*x11^3 - p31*x15 - p43*x15 - p49*p25*x15 .

d(x16)/dt = (x17*p41)/2 - x16*p46 .

d(x17)/dt = p34*p35*(p37 + (p38*x20^p39)/(p40^p39 + x20^p39)) - x17*p45 - x17*p41 .

d(x18)/dt = x10*p13 - x18* p50 + x16*p42 - x8*x18*p12 + x10*p20*p9 + x10*p19*p11 .

d(x19)/dt = 2*p27*x11 - p28*x19 - p32*x19 + 2*p25*x20 - p43*x19 + p43*x20 + 2*p44*x20 + 2*p43*x21 - 3*p26*p24*x19^3 + p33*p32*x20 + 3*p49*p25*x21 - 2*x23*p24*x19^2 .

d(x20)/dt = x23*p24*x19^2 + 2*p31*x14 - p25*x20 - p43*x20 - p44*x20 - p33*p32*x20 .

d(x21)/dt = p26*p24*x19^3 + 2*p31*x15 - p43*x21 - p49*p25*x21 .

d(x22)/dt = 2*x12*p27 - x22*p28 - x22*p43 + p32*x19 + p33*p32*x20.

d(x23)/dt = - x23*p24*x19^2 + 2*x13*p29 - x23*p30 - x23*p44 + p25*x20 + p43*x20 + p33*p32*x20 .

For convenience of space, the 23 species in the model were represented by the symbols xi. Appendix Table S2 summarizes how these species were termed in Figure 4B and provides a short description.

Appendix Table S2: List of model species

Symbol	Name in Fig. 4B	Description
x1	R1	TGFβ receptor I -cell surface
x2	R2	TGFβ receptor II -cell surface
x3	R1e	TGFβ receptor I- endosomal
x4	R2e	TGFβ receptor II –endosomal
x5	TGFβ	Free TGFβ ligand –medium
x6	TGFβ_In	TGFβ ligand –endosomal
x7	R2L	Complex of TGFβ and its receptor TGFβ receptor II -cell surface
x8	R1R2-L	Activated complex of R1 and R2L - cell surface
x9	R1R2-L-e	Activated complex of R1 and R2L – endosomal
x10	Inactive_Rec	Complex of R1R2-L with the feedback regulator (FB)-cytoplasmic
x11	pS2	phosphorylated SMAD2 –cytoplasmic
x12	S2	unphosphorylated SMAD2 –cytoplasmic
x13	S4	SMAD4 –cytoplasmic
x14	pS2_pS2_S4	heterotrimer of two phosphorylated SMAD2 and one SMAD4 –cytoplasmic
x15	pS2_pS2_pS2	homotrimer of phosphorylated SMAD2 –cytoplasmic
x16	FBmRNA	mRNA of the feedback element(FB) - cytoplasmic
x17	FBmRNA1	mRNA of the feedback element(FB) - nuclear
x18	FB	Feedback protein(s), present both basaly and induced upon TGFβ stimulation
x19	pS2n	phosphorylated SMAD2 –nuclear
x20	pS2_pS2_S4n	heterotrimer of two phosphorylated SMAD2 and one SMAD4 –nuclear
x21	pS2_pS2_pS2n	homotrimer of phosphorylated SMAD2 –nuclear
x22	S2n	unphosphorylated SMAD2 –nuclear
x23	S4n	SMAD4 –nuclear

Appendix Table S3: List of model parameters

Description and alternative naming (used in Appendix Table S5) for the 50 model parameters represented by the symbols pi.

Symbol	Parameter name	Description
p1	k_R1_production	Production rate of R1
p2	k_R2_production	Production rate of R2
p3	k_S2_production	Production rate of S2
p4	k_S4_production	Production rate of S4
p5	kf_R2_activation	TGFβ (L) and R2 binding rate
p6	kb_R2_activation	L and R2 unbinding rate
p7	kf_R1_activation	R2L binding rate to R1
p8	kb_R1_activation	R2L unbinding rate to R1
p9	kdeg_R2	R2 degradation rate
p10	kdeg_R1	R1 degradation rate
p11	kin_deg_Ligand	Endosomal ligand degradation rate
p12	kf_Seq_FB_Rec	R1R2-L binding rate with FB
p13	kb_Seq_FB_Rec	R1R2-L unbinding rate with FB
p14	k_in_1	Influx rate R1, surface -> endosome
p15	k_in_2	Influx rate R2, surface -> endosome
p16	index_k_out_1_relative_speed	Relative out flux of R1, scaled by its influx
p17	index_k_out_2_relative_speed	Relative out flux of R2, scaled by its influx
p18	index_active_Rec_internalize	Influx rate R1R2-L, surface -> endosome
p19	index_induced_ligand_deg	Ligand degradation induced by FB
p20	index_induced_R2_deg	R2 degradation induced by FB
p21	k_disso_Active_Rec	Unbinding rate of R1R2-L-e
p22	k_phosphorylation	S2 phosphorylation rate
p23	effective	Receptor inhibition strength
p24	kf_trimmer	Binding rate, trimer formation
p25	kb_trimmer	Unbinding rate, trimer formation
p26	index_kf_homotrimer	Homotrimer formation rate, scaled by trimer formation rate
p27	S2_import_to_nuc	S2 nuclear import rate
p28	S2_export_from_nuc	S2 cytoplasmic export rate

p29	S4_import_to_nuc	S4 nuclear import rate
p30	S4_export_from_nuc	S4 cytoplasmic export rate
p31	Trimer_import_to_nuc	Trimer nuclear import rate
p32	k_Dephos	pS2 dephosphorylation rate
p33	index_trimer_dephos	Trimer dephosphorylation rate scaled to pS2 dephosphorylation rate
p34	DRB_index	Strength of DRB mediated transcriptional inhibition
p35	FB_KD_index	Extent of FB knockdown in the model upon experimental Smad7 KO.
p36	Rec_inhibitor	Kept zero for all dataset except receptor inhibition dataset where it is 1.
p37	mRNA_prod	Basal production rate of FB mRNA
p38	k_induced_FB_production	Induced production rate of FB mRNA
p39	hill_fact1	Hill coefficient of transcriptional induction of FB mRNA
p40	K_mrna	Km of transcriptional induction of FB mRNA
p41	export_cytoplasm	Cytoplasmic export rate of the FB mRNA
p42	k_FB_protein	FB protein production rate
p43	kdeg_S2	S2 degradation rate
p44	kdeg_S4	S4 degradation rate
p45	kmRNA1deg_FB	Nuclear degradation rate of FB mRNA
p46	kmRNAdeg_FB	Cytoplasmic degradation rate of FB mRNA
p47	BolusInjection1	Step function for ligand injection
p48	k_medium	Medium volume
p49	index_kb_homotrimer	Unbinding rate of homotrimer, scaled to unbinding rate of trimer
p50	kdeg_FB	Degradation rate of FB protein

The ODEs contain four synthesis rates for R1, R2, S2 and S4 (parameters p1-p4). We found it more intuitive to formulate the system using the total concentrations of these species instead of synthesis rate. To this end, we analytically solved the steady state of the ODEs (dxi/dt = 0) in the absence of TGFβ, and obtained algebraic terms by which p1-p4 can be replaced and thereby removed from the system of ODEs.

p1 = (R1_total * (p14*(p10)^2)/((p16 * p14) * p10 + p10^2 + p14*p10)) .

p2 = (R2_total * p15*(p9)^2)/((p17 * p15) * p9 + p9^2 + p15*p9)) .

p3 = (S2_total * ((p27*p43 + p28*p43 + p43^2)/((p28 + p43) + 2*p27)) .

p4 = (S4_total * ((p29*p44 + p30*p44 + p44^2)/((p30 + p44) + 2*p29)) .

III.C. Model simulation

The model described above was solved numerically using the Matlab toolbox Data2Dynamics (Raue *et al*, 2015). In the following, it will be described which initial conditions were used for these numerical simulations and how experimental perturbations were implemented.

Initial conditions: The unmodified protein species in the model (e.g., unphosphorylated SMAD2 or unbound receptors) are expressed at non-zero, basal levels even in the absence of stimulation. The initial levels of these proteins are determined by their synthesis and degradation rates, and these parameters are subject to change during model fitting (see below). Furthermore, S2 and S4 shuttle between cytoplasm and nucleus, and the receptors (R1, R2) between plasma membrane and endosome (Vizán *et al*, 2013; Schmierer *et al*, 2008). The protein distribution between these compartments is again determined by the fitted shuttling parameters. To correctly assign the initial concentrations of the corresponding model species, we analytically calculated their steady state levels (dxi/dt = 0) in the absence of TGFβ. The following equations describe the steady state (SS) solutions for S2, S4, R1, R2, FB mRNA as well as FB protein (FB).

R1_SS = ((R1_total * (p14 * (p10)^2)/((p16 * p14) * p10 + p10^2 + p14 * p10)) * ((p16 * p14) + p10))/(p14 * p10) .

R2_SS = ((R2_total * (p15 * (p9)^2)/((p17 * p15) * p9 + p9^2 + p15*p9))*((p17 * p15) + p9))/(p15 * p9) .

R1e_SS = (R1_total * (p14 * (p10)^2)/((p16 * p14) * p10 + p10^2 +p14 * p10))/p10 .

R2e_SS = (R2_total * (p15*(p9)^2)/((p17 * p15) * p9 + p9^2 + p15 * p9))/p9 .

S2_SS = ((S2_total * ((p27 * p43 + p28 * p43 + p43^2)/((p28 + p43) + 2 * p27))) * (p28 + p43))/(p27 * p43 +p28 * p43 + p43^2) .

S4_SS = (S4_total * ((p29 * p44 + p30 * p44 + p44^2)/((p30 + p44) + 2 * p29))) * (p30 + p44))/(p29 * p44 +p30 * p44 + p44^2) .

S2n_SS = (2 * p27 * (S2_total * ((p27*p43 + p28*p43 + p43^2)/((p28 + p43) + 2 * p27))))/(p27 * p43 + p28 * p43 + p43^2) .

S4n_SS = (2 * p29 * (S4_total * ((p29 * p44 + p30 * p44 + p44^2)/((p30 + p44) + 2 * p29))))/(p29 * p44 + p30*p44 + p44^2) .

FBmRNA1_SS = (p41 * p37)/(2 * p46 * (p41 + p45)) .

FBmRNA_SS = p37/(p41 + p45) .

FB_SS = (p41*(FB_total * (2 * p50 * p46 * (p41 + p45)))/(p41 * p37)) * p37)/(2 * p50 * p46 * (p41 + p45)) .

Modeling of experimental perturbations: We experimentally analyzed the dynamics of the signaling pathway under various experimental perturbations and in the following will describe how these perturbations were implemented in the model.

TGFβ stimulation: In our model, TGFβ is subject to degradation due to internalization into cells, implying that an added amount of ligand does not stay constant, but decays over time (see Figure 4D). We therefore did not assume an excess of external ligand, but added a certain amount of TGFβ using a step function at t = 0.
Here the step function used is scaled for desired output. The step function multiplied by a value of s1 = 0.47 results in a TGFβ signal of 1 pM and successive doses strengths can be achieved by introducing linear change in s1. For instance, s1 = 47 multiplied to the step function gives an output of 100pM TGFβ.

TGFβ restimulation: In the experimental setup for restimulation, TGFβ is added to the medium after removing the ligand remaining from a previous treatment (see Figure EV4D). Restimulation was implemented in the model by washing out the ligand from the initial stimulus (see above) by setting medium TGFβ to zero at the time point of restimulation, and applying a second step function stimulus of desired height.

TGFβ receptor inhibition: We applied the TGFβ R1 kinase inhibitor SB431542 which specifically and rapidly terminates the TGFβ signaling (Inman & Hill, 2002) (see Figure EV4E-F). Receptor inhibition is implemented by multiplying the flux equation of the S2 phosphorylation step (S2_c => pS2_c)" with the function (1 - Rec_inhibitor * effective). Here Rec_inhibitor is a step function that can be turned on (from 0 to 1) at the time of inhibition. To accommodate that the receptor inhibition may not have an efficiency of 100%, we allowed a factor "effective" that was allowed to vary in the range from 0-1 during model fitting to the experimental data (see Appendix Table S3).

DRB pretreatment: To validate the presence of transcriptional feedback loops in TGFβ signaling we conducted experiments in presence of DRB, a RNA Polymerase II inhibitor that globally and rapidly blocks mRNA transcription (Figure 4H). The cells were treated with DRB 15 minutes or 60 minutes prior to TGFβ stimulation. DRB treatment was modeled using a step function that enters the mRNA production reaction for the feedback loop (=> FBmRNA1) as the "DRB_index" term (see Appendix Table S3). The DRB_index is 1 for the data sets with no DRB treatment and switched to a value between 0 (complete block) and 1 (no block) at 15 or 60 min prior to TGF stimulation for DRB treatment simulations. Because literature evidence suggesting that some genes may still be transcribed upon DRB treatment (Bensaude, 2011) we varied DRB_index in the range [0-1, (0 = full inhibition and 1 = no inhibition)] and simulated the model keeping the rest of the model parameters constant and found a best-match for DRB_index = 0.2 which indicates an 80% reduction in the strength of feedback loop. Furthermore, we observe residual TGFβ-induced transcription in the presence of DRB.

SMAD7 knockout: A functional SMAD7 knockout was experimentally introduced in MCF10A cells by truncating the protein such that it can no longer bind and inhibit the TGFβ receptor complex (Figure EV5E-F, Materials and Methods). In the model, this knockout was implemented by simultaneously reducing the basal and induced mRNA production of the generic feedback regulator (FB) by the same factor (“FB_KD_index” in Appendix Table S3). Some feedback was assumed to remain in the knockout condition (FB_KD_index > 0), since TGFβ induces expression of several negative feedback regulators that act at the receptor level (e.g., BAMBI, STRAP, FKBP12) and are

functionally redundant with SMAD7 (Massagué & Chen, 2000; Shi & Massagué, 2003; Legewie *et al*, 2008). The residual FB strength in the SMAD7 knockout condition was systematically estimated by comparing the simulation of the best-fit model for varying FB_KD_index to the experimental data from SMAD7 knockout cells (see Section III.D).

III.D Model calibration

The kinetic parameters of the model described above were unknown, and had to be estimated by fitting the model trajectories to experimental data. In this section it will be described which datasets were used for fitting and how fitting was implemented.

Model calibration datasets**:** The model was calibrated by simultaneously fitting to several datasets. Most of these measurements characterize the nucleocytoplasmic shuttling of SMAD2 or SMAD4 using live-cell imaging, the only exceptions being Western Blot measurements of the total TGFβ receptor concentrations (dataset 6) and a qPCR analysis of SMAD7 mRNA expression (dataset 7). The dataset described are summarized in Appendix Table S4.

Appendix Table S4: Datasets used for calibration of population-average model

#	Name	Observable Species	TGFβ concentrations	Additional perturbations	Observation time (Sampling rate)	Figure
1	SMAD2 translocation dynamics - TGFβ titration	Median SMAD2-YFP nuc/cyt-ratio	1, 2.5, 5, 25 and 100pM	None	24h (5 min)	4C
2	SMAD4 translocation dynamics - TGFβ titration	Median SMAD4-YFP nuc/cyt-ratio	1, 2.5, 5, 25 and 100pM	None	24h (5 min)	EV4A
3	SMAD2 translocation dynamics - Restimulation (2.5pM TGFβ)	Median SMAD2-YFP nuc/cyt-ratio	repeated stimulation with 2.5 pM TGFβ (0+3h and 0+6h, respectively)	None	24h (5 min)	EV4D
4	SMAD2 translocation dynamics - Receptor inhibition (100pM TGFβ)	Median SMAD2-YFP nuc/cyt-ratio	100pM	TGFβRI inhibitor SB431542 applied early (1.5h) or late (6h) after TGFβ stimulation	24h (5 min)	EV4E
5	SMAD4 translocation dynamics - Receptor inhibition (100pM TGFβ)	Median SMAD4-YFP nuc/cyt-ratio	100pM	TGFβRI inhibitor SB431542 applied early (1.5h) or late (6h) after TGFβ stimulation	24h (5 min)	EV4F
6	Total receptor expression (100pM TGFβ)	immunoblots of total cellular TGFβ receptors 1 and 2	100pM	None	8h (1hr until 4h; 2h from 4h to 8h)	EV4B-C
7	S7mRNA time course – TGFβ titration	S7mRNA expression (RT-PCR)	2.5, 5, 25 and 100pM	None	24h (0.75 1.5 3 6 8 14 24)*h	EV4G

Optimization: The model simulations were fitted by adjusting certain species in the model (or combinations thereof) to the corresponding experimental data. This section describes in detail how model and data were compared during optimization.

Observables: As summarized in Appendix Table S4, the model was simultaneously fitted to the population-median nucleo-cytoplasmic (n/c) ratios of SMAD2 and SMAD4 (imaging), the fold-change in TGFβ receptor 1 and 2 protein expression (immunoblotting) and the fold-change in SMAD7 mRNA expression (qPCR). The species in the model were combined into the following observables to compare the model simulation with the experimental measurements (nomenclature according to section in III.B)

SMAD2(n/c) = ((2 * x20) + x19 + x22 + 3 * x21)/((x11 + x12 + (2 * x14) + (3 * x15))) .

SMAD4(n/c) = (x20 + x23)/((x14 + x13)) .

Rec1 = ((x1 + x3 + x8 + x9 + x10)/(R1_Sl +lR1e_SS)) .

Rec2 = ((x2 + x4 + x7 + x8 + x9 + x10)/(R2_SS + R2e_SS)) .

S7mRNA = ((x16 + x17)/(x16 + x17)) .

The value of SMAD2(n/c) from the model, for instance, was compared to the experimentally measured nucleocytoplasmic ratio at the respective time points. Optimization was carried out using the MATLAB optimization toolbox Data2Dynamics (Raue *et al*, 2015). The goodness of fit is calculated here by evaluating a log likelihood function which comprises of residual that minimizes the difference between model and data as well as the contribution from the fitted error models specific to the different data sets. Details of its implementation can be found elsewhere [31],[35]. Fitting was done using a multi-start local optimization strategy where the starting parameter vectors were generated using Latin hypercube sampling and it uses the MATLAB function lsqnonlin for optimization which minimizes the sum of residuals (Raue *et al*, 2013).

Offsets and scaling factors: It is commonly observed that biological replicates collected are qualitatively similar, but exhibit quantitative differences. Similarly, we observed slightly different values in the nucleocytoplasmic SMAD ratio, e.g., in the basal and maximal values after stimulation. During fitting, we introduced an offset and a scaling

factor specific for the day of measurement to accommodate these effects and to quantitatively fit datasets 3-8 above, whereas no scaling factors were assumed for datasets 1 and 2.

Chi-square = (offset + scale * model - data)^2/error^2

The best-fit values of these scaling factors offsets and the data-specific error model parameters are given in Appendix Table S5B. To this end we have 45 parameters from the model (Appendix Table S5A), and 34 other parameters which includes constants that are multiplied to step functions for obtaining desired signal as well three other types of fitted parameters (1 - offsets 2 - scaling factors and 3 - error model parameters), all of which are shown in Appendix Table S5B. It can be noted that there are 50 parameters listed in Appendix Table S3 of which the dataset specific parameters "DRB_index", "FB_KD_index", "Rec_inhibitor", "effective" and a common parameter " BolusInjection1" (for the input step function) are not shown in Appendix Table S5A.

Including offsets and scaling factors, the model had 72 free parameters that had to be estimated by fitting, while constraining them to a physiologically reasonably range based on previous studies (Appendix Table S5A). Parameters for additional 30 independent model fits with similar goodness-of-fit obtained from local multi-start optimization can be found in Appendix Table EV1, their distributions in Appendix Figure S4.

Appendix Table S5A: Best-fit model parameters of the population-average model

Parameter	best-fit value	lower bound	upper bound	units
'K_mran'	223.5	0.1	5000	nM
"R1_total'	34.8	3	35	nM
'R2_total'	34.9	3	35	nM
'S2_export_From_nuc'	1.99	0.03	2	(min)-1
'S2_import_to_nuc'	0.37	0.03	2	(min)-1
'S2_total'	875.9	10	1000	nM
'S4_export_From_nuc'	0.196	0.03	2	(min)-1
'S4_import_to_nuc'	0.037	0.03	2	(min)-1
'S4_total'	629.9	10	1000	nM
'Trimer_import_to_nuc'	0.14	0.03	2	(min)-1
'export_cytoplasm'	0.03	0.03	2	(min)-1
'hill_fact1'	3.98	1	10	none
'index_active_Rec_internalize'	0.54	0.5	3	none
'index_induced_R2_deg'	1.0	1	4	none
'index_induced_ligand_deg'	2.72	1	4	none
'index_k_out_1_relative_speed'	0.56	0.15	1	none
'index_k_out_2_relative_speed'	0.24	0.15	1.9	none
'index_kb_R1'	1.61	0.01	10	none
'index_kb_R2'	8.70	0.01	10	none
'index_kb_homotrimer'	0.0163921523	0.0001	10	none
'index_kf_homotrimer'	0.651839125	0.001	10	none
'index_seq_kb'	0.001	0.00001	10	none
'index_trimer_dephos'	2.61	1	5	none
'k_Dephos'	0.14	0.001	0.5	(min)-1
'k_FB_protin'	0.22	0.001	1	(min)-1
'k_disso_Active_Rec'	0.001	0.001	2	(min)-1
'k_in_1'	0.2007	0.01	10	(min)-1
'k_in_2'	0.625	0.01	10	(min)-1
'k_induced_FB_production'	4.99994	0.01	5	(min)-1
'k_medium'	2018.92	2000	2200	μl
'k_phosphorylation'	0.070	0.001	5	(min)-1
'kb_trimmer'	1.607	0.001	50	(min)-1
'kdeg_R1'	0.058	0.001	0.06	(min)-1
'kdeg_R2'	0.001	0.001	0.06	(min)-1
'kdeg_S2'	0.0005	0.0005	0.005	(min)-1
'kdeg_S4'	0.0048	0.0005	0.005	(min)-1
'kdeg_FB'	0.001	0.001	0.3	(min)-1
'kf_R1_activation'	4.985	0.01	5	(nM)-1.(min)-1
'kf_R2_activation'	4.955	0.001	5	(nM)-1.(min)-1
'kf_Seq_FB_Rec'	0.882	0.03	1	(nM)-1.(min)-1
'kf_trimmer'	4.814	0.001	5	(nM)-1.(min)-1
'kin_deg_Ligand'	0.720	0.05	0.8	(min)-1
'kmRNA1deg_FB'	0.005	0.005	0.1	(min)-1
'kmRNAdeg_FB'	0.099	0.005	0.1	(min)-1
'mRNA_prod'	0.013	0.001	1	(min)-1

Appendix Table S5B: Offsets, scaling factors and error parameters of the best-fit model

Parameter	best-fit value	lower bound	upper bound	Relevance
'k1'	0.47	none	none	get 1pM TGFβ
'k2'	1.175	none	none	get 2.5pM TGFβ
'k3'	2.35	none	none	get 5pM TGFβ
'k4'	11.75	none	none	get 25pM TGFβ
'k5'	47	none	none	get 100pM TGFβ
'k2_restimulation'	1.175	none	none	get 2.5pM TGFβ, restimulation
'kS2_effective_1'	0.85	0.6	1(1 =100% effective)	get efficiency of Rec Inhibitor; SMAD2, 1.5hr dataset
'kS2_effective_2'	0.99	0.6	1(1 =100% effective)	get efficiency of Rec Inhibitor; SMAD2, 6hr dataset
'kS4_effective_1'	1	0.6	1(1 =100% effective)	get efficiency of Rec Inhibitor; SMAD4, 1.5hr dataset
'kS4_effective_2'	1	0.6	1(1 =100% effective)	get efficiency of Rec Inhibitor; SMAD4, 6hr dataset
offset_DRB_100_old'	0.28	0.2	5	DRB at TGFβ = 100pM, WT
'offset_S2_Rec_Ini'	1.09	0.2	5	offset
'offset_S4_Rec_Ini'	1.77	0.2	5	offset
'offset_S7_mRNA'	0.94	0.2	5	offset
'offset_restimulation'	0.34	0.2	5	offset
'scale_DRB_100_minus_60min'	0.86	0.2	5	scaling factor
'scale_S2_Rec_Ini'	0.94	0.2	5	scaling factor
'scale_S4_Rec_Ini'	0.62	0.2	5	scaling factor
'scale_S7_mRNA'	1.3	0.2	5	scaling factor
'scale_restimulation'	0.35	0.2	5	scaling factor
'sd_Rec1'	2.5	0.005	5	estimated error parameter
'sd_Rec2'	1.99	0.005	5	estimated error parameter
'sd_S2_Rec_ini'	0.024	0.005	5	estimated error parameter
'sd_S4_Rec_ini'	0.048	0.005	5	estimated error parameter
'sd_S4_WT'	0.024(WT)	0.005	5	estimated error parameter
'sd_S4_Rec_Ini'	0.03(REC_INI)	0.005	5	estimated error parameter
'sd_S7_mRNA'	0.94	0.005	5	estimated error parameter
'sd_pS2_S2_WT'	0.0393968609(WT)	0.005	5	estimated error parameter
'sd_pS2_REc_Ini_WT'	0.0503800121	0.005	5	estimated error parameter
'sd_pS2_restimulation_WT'	0.0266884823	0.005	5	estimated error parameter
'sd_pS2_restimulation_3hr'	0.0222209887	0.005	5	estimated error parameter
'sd_pS2_restimulation_6hr'	0.0257695112	0.005	5	estimated error parameter
'sd_pS2_DRB_WT'	0.1195361719	0.005	5	estimated error parameter
'time1_2_5'	10	none	none	Ligand injection time, restimulation, 2.5pM

III.E. Model validation

The best-fit model as well as 30 independent model fits with similar goodness-of-fit obtained from local multi-start optimization were validated by experimentally testing their predictions for conditions not used for model calibration. The validation data sets are summarized in Appendix Table S6.

Table S6: Datasets used for validation of population-average model

#	Name	Observable Species	Conditions	Observation time (Sampling rate)	Figure	Main observation for model validation
1	TGFβ degradation	extracellular TGFβ	25pM	24h (every 1h for 1 to10 h and [13, 14, 16, 18, 24]h.	4D	complete depletion of 25 pM TGFβ occurs at ~ 20h, and coincides with signaling termination
2	SMAD2 translocation dynamics - Restimulation (5 pM TGFβ)	median nuc/cyt SMAD2-YFP ratio	repeated stimulation with 5pM (0+3h, 0+8h)	24h (5 min)	4E (0+3h)<br>4F (0+8h)	pathway restimulation more effective at late time points for low TGFβ concentrations.
3	SMAD2 translocation dynamics - Restimulation (100pM TGFβ)	median nuc/cyt SMAD2-YFP ratio	repeated stimulation 100pM (0+3h, 0+8hr)	24h (5 min)	EV4G (0+3h)<br>4G (0+8h)	pathway restimulation is ineffective at early and late time points at high TGFβ concentrations.
4	SMAD2 translocation dynamics – DRB pre-treatment	median nuc/cyt SMAD2-YFP ratio	treatment with DRB 60 minutes prior to TGFβ stimulation	6h post stimulation (5min)	4H	nuc/cyt SMAD2 ratio is enhanced upon DRB pre-treatment supporting the presence of transcriptional negative feedback.

The DRB pre-treatment experiment (#3) was used to confirm our finding that the model could only fit the calibration data sets (Appendix Table S4) if a transcriptionally induced negative feedback loop via the generic species FB was included. To quantitatively compare the simulations of the best-fit model with the DRB experiment, we systematically varied the "DRB_index", which changes the degree of transcriptional inhibition by DRB (Appendix Table S3). The closest match between the measured median nuc/cyt-ratio upon DRB treatment and the simulation was observed for DRB_index = 0.2, indicating an 80% inhibition of transcription by DRB (Section III.C). Please note that we first quantitatively adjusted the best-fit model to the untreated control of the DRB experiment using offset and scaling factor to accommodate minor quantitative differences between the SMAD2 nuc/cyt-ratios at different days.

The ligand depletion (#1) experiment confirmed our model prediction that cell-

mediated ligand degradation controls the duration of SMAD signaling, especially at low TGFβ doses. As a consequence of integrating transcriptional feedback and TGFβ degradation as two main adaptation mechanisms our model could correctly describe the refractoriness of the pathway towards restimulation (#2).

Taken together, the model successfully predicts novel experimental perturbations, and therefore seems to capture the main mechanisms shaping the SMAD signaling dynamics.

IV. Quantitative modeling of signaling heterogeneity

The population-median model described in the previous sections captured the dynamic behavior of an average cell. To quantitatively describe the cell-to-cell variability in the signaling pathway (Figure 2A), we adopted a deterministic modeling approach in which the cell-population is described by an ensemble of heterogeneous single-cell models. In line with previous studies (Kallenberger *et al*, 2014; Spencer *et al*, 2011; Carpenter *et al*, 2006), we assumed that signaling heterogeneity arises from cell-to-cell variability in signaling protein expression. As described in the following, we introduce a novel tiered approach for a quantitative description of heterogeneity (Figure 4A) in which we initially derive six sub-population models and then describe the complete cell population by sampling the total signaling protein concentration from log-normal distributions in each sub population model.

IV.A Derivation of six sub-population models

Our clustering analysis of the SMAD nuclear translocation time courses suggested that the cell population consists of six sub-populations with qualitatively distinct dynamic behavior (Figure 2C and 2D). We converted the best-fit population-average model as well as 30 independent model fits with similar goodness-of-fit obtained from local multi-start optimization into six sub-population models each by separately fitting the models to the median SMAD2 nucleocytoplasmic ratio of each cluster at a dose of 100 pM TGFβ. Therefore, these subpopulation models are also termed "cluster models" below. Differences between the subpopulation models were introduced by allowing the signaling protein concentrations to vary within a narrow range (0.5-2 fold of best-fit population-average parameter value, Appendix Table S5A) during fitting to mimic that protein may differ between clusters. The remaining kinetic parameters in the model were assumed to be the same for all clusters with the exception of a few kinetic parameters describing lumped chemical reactions that are themselves a function of several intracellular protein concentrations: For instance, the nuclear import and export of SMAD2 and SMAD4 is carried out by specific nucleoporins such as RanBP3 and CAN/Nup214 (Xu *et al*, 2012; Dai *et al*, 2009). Similarly, the shuttling of TGFβ receptors between plasma membrane and endosomal compartments is controlled by clathrin and related adapter proteins (Mitchell *et al*, 2004). By allowing heterogeneity in these steps, we accommodate that sub-populations (or single-cells) might have different initial

distributions of SMAD2 and TGFβ receptors between sub cellular compartments (compare main figure 2A). Appendix Table S7 lists all protein concentrations and kinetic parameters that were allowed to change during sub-population model fitting and shows their best-fit values. Parameters for the additional 30 signaling class fits obtained from independent population-average model fits are provided in Appendix Table EV1.

Appendix Table S7: Best-fit parameter values of the six sub population models.

Parameter	Population -average best-fit	Cluster 1 best-fit	Cluster 2 best-fit	Cluster 3 best-fit	Cluster 4 best-fit	Cluster 5 best-fit	Cluster 6 best-fit
R1_total	34.87	19.07	49.64	69.69	34.51	69.75	43.36
R2_total	34.99	17.6	23.5	20.5	19.7	63.1	66.8
S2_export_from_nucleus	1.99	2.0	2.8	3.9	2.9	2.5	1.5
S2_import_to_nucleus	0.37	0.4	0.38	0.18	0.56	0.23	0.37
S2_total	875.9	1625.4	1231.9	717.6	714.3	470.9	1250.3
S4_export_from_nucleus	0.19	0.18	0.09	0.1	0.1	0.39	0.39
S4_import_to_nucleus	0.038	0.037	0.074	0.074	0.018	0.021	0.018
S4_total	629.9	904.5	672.7	314.9	345.3	316.1	314.9
k_FB_protein	0.22	0.43	0.11	0.11	0.19	0.12	0.26
index_active_Rec_internalize	0.54	0.3	0.26	1.07	1.07	0.27	1.07
index_k_out_1_relative_speed	0.57	0.38	1.12	1.13	0.3	1.12	1.13
index_k_out_2_relative_speed	0.24	0.12	0.49	0.49	0.12	0.49	0.18
k_Dephosphorylation	0.14	0.07	0.27	0.069	0.069	0.07	0.07
k_in_R1	0.2	0.16	0.1	0.4	0.4	0.2	0.36
k_in_R2	0.62	1.18	1.25	1.13	1.25	0.35	1.08
k_induced_FB_production	4.99	9.75	2.5	2.5	8.58	2.73	3.97
mRNA_prod_FB	0.013	0.025	0.007	0.026	0.009	0.01	0.02

Assumption of spatially homogeneous ligand degradation: During cluster fitting, each cluster model contains a different set of protein concentrations and kinetic parameters (Appendix Table S7). Since TGFβ degradation depends on receptor endocytosis, each sub-population will degrade the ligand with different kinetics, and may, in principle, experience a locally different TGFβ concentration. We assumed TGFβ to diffuse fast within the cell culture dish and therefore neglected such spatial heterogeneity in TGFβ concentrations. During fitting, we imposed that each subpopulation experiences the population-average ligand concentration by: (i) choosing a very high medium volume, implying that each cell (or sub-population) negligibly contributes to the total degradation of the ligand; (ii) during cluster fitting, a new overall ligand degradation reaction with linear kinetics is added that mimics the ligand decay in the population-average model (TGFβ =>, flux equation "kout_deg_Ligand_100pM * TGFβ"). The rate constant "kout_deg_Ligand_100pM" was determined by fitting the trajectory of this

apparent ligand degradation reaction to the ligand decay time course of the population-average model for 100pM TGFβ, and was kept same for all the clusters. This implementation reproduces the population-average decay for 100pM and ensures that all cluster models experience the same global TGFβ concentration over time. Similarly, while simulating the single cells for lower doses of TGFβ, for instance 2.5pM, the trajectory of ligand decay from the population average model was fitted to obtain the linear decay rate for that given dose. This way we could introduce the dose specific ligand decay in the cluster models and all this was done in the population average model by keeping the already fitted model parameters fixed coupled to conditions (i) and (ii) above.

IV.B Simulation of a complete cell population (cluster model approach)

As a next step, we employed the six best-fit cluster models representing the signaling subpopulations at 100 pM TGFβ to simulate a complete cell population comprising thousands of cells. To this end, repeated simulations were performed for each cluster model, and it was assumed that each simulation run represents a single cell. Cell-to-cell variability was introduced by sampling the above-mentioned protein concentrations and lumped parameters (Appendix Table S7) from log-normal distributions for each simulation run. Hence, the expression noise of signaling proteins was assumed to follow a lognormal distribution which is in line with published literature studies (Jeschke *et al*, 2013; Sigal *et al*, 2006), and with our own quantification of cytosolic SMAD2 and SMAD4 concentrations in unstimulated cells. To obtain good statistics 10.000 cells were simulated for each cluster and the complete cell population was assembled by combining cells from all clusters, each cluster entering in the proportion that we had observed in the original clustering of the 100pM TGFβ data (Total number of cells: 352; cluster 1: 10 cells; cluster 2: 14 cells; cluster 3: 39 cells; cluster 4: 146 cells; cluster 5: 62 cells; cluster 6: 81 cells). To combine the cluster simulations into a complete population of 10.000 cells, we take weighted fraction of cells from each simulated cluster: 10.000*(number of cells in cluster i)/(total number of cells). For example, the number of contributing cells from cluster 1 is given by (10/352) * 10000 = 284.1 cells, and this number is rounded to the nearest integer.

Matching simulated and measured heterogeneity by noise titration: In the

subpopulation modeling step (Section III.A), we already introduced some heterogeneity in the first step by allowing different protein concentrations in the fit to each signaling cluster. The degree of variability that additionally had to be introduced while simulating the complete cell population was unknown. To optimize the model-based description of signaling heterogeneity, we systematically tested different noise levels in the single-cell sampling step and asked which noise level quantitatively reproduces the experimentally measured heterogeneity in SMAD2 nuclear translocation. For simplicity, we assumed that all signaling protein concentrations exhibit the same degree of variability around their mean value, and that the variability of each protein is the same for all clusters. We further presumed that the protein expression noise consists of two proportions, an extrinsic (correlated) and an intrinsic (uncorrelated) component. These two components are well-described in the gene expression noise literature (Rhee *et al*, 2014; Swain *et al*, 2002): each promoter (of a gene encoding for a signaling protein) is present in low copy numbers and this gives rise to stochasticity in mRNA expression that is specific for each gene (uncorrelated component). At the same time, protein expression is controlled by factors reflecting the global state of the cell. For instance, a cell might contain high copy numbers of polymerase II or ribosomes, and this will simultaneously affect the expression of all signaling proteins, thereby giving rise to correlated fluctuations. Correlated and uncorrelated noise contributions were sampled from log-normal distributions using the MATLAB lognrnd function. For each protein xj with a population-average concentration Xj, the protein in cell i is given by $xj_{(i)}$ = Xj * Correlated noise * lognrnd(0, noise2), where Correlated_noise = lognrnd(0, noise1) is shared for all proteins in the same cell.

To test for different correlated and uncorrelated noise contributions, we scanned a two-dimensional grid, and assumed the following standard deviations noise1 and noise2 are varied as

noise1 = [0.01, 0.05, 0.1, 0.125, 0.15, 0.175, 0.2, 0.22, 0.25, 0.27, 0.3] and
noise2 = [0.01, 0.05, 0.1, 0.125, 0.15, 0.175, 0.2, 0.22, 0.25, 0.27, 0.3];

In order to determine the optimal combination of correlated and uncorrelated noise contributions, we compared the model simulation to the experimental data. To this end, we defined signaling features for each single cell that define the dynamics of the SMAD2 nucleocytoplasmic ratio and assessed their heterogeneity. The signaling features

compared between model and data are schematically depicted in Figure 5B and defined as

1. Early peak amplitude (E), calculated as the median S2 N/C-ratio between 50 to 75 minutes
2. Late plateau (L), calculated as the median S2 N/C-ratio between 290 to 310 minutes
3. The ratio E/L as a measure of signal adaptation after the initial peak
4. Basal levels (B), calculated as S2 N/C-ratio at before stimulation (0 min)
5. The coefficient of variation (CV=std/mean) of model and data for features mentioned in 1-4.

The distributions of B, E, L, and E/L were calculated from the population of 10000 simulated cells and the area under the histogram was normalized to match the histogram of 352 measured cells at 100pM TGFβ in order to overlay simulated and measured distributions (Figure 5E). A quantitative comparison for each correlated/uncorrelated noise combination was done by calculating a cost function that sums up the differences between of the simulated and measured distributions at each of the N bins of the histograms.

$$CostAmplitude_{(correlatedNoise,uncorrelatedNoise)} = \left|\sum_{i=1}^{N} \mathrm{Amplitude}_{(B_i[\mathrm{model}])} - \mathrm{Amplitude}_{(B_i[\mathrm{data}])}\right| + \left|\sum_{i=1}^{N} \mathrm{Amplitude}_{(E_i[\mathrm{model}])} - \mathrm{Amplitude}_{(E_i[\mathrm{data}])}\right| + \left|\sum_{i=1}^{N} \mathrm{Amplitude}_{(L_i[\mathrm{model}])} - \mathrm{Amplitude}_{(L_i[\mathrm{data}])}\right| + \left|\sum_{i=1}^{N} \mathrm{Amplitude}_{(E_i/L_i[\mathrm{model}])} - \mathrm{Amplitude}_{(E_i/L_i[\mathrm{data}])}\right|.$$

$$CostCV_{(correlatedNoise,uncorrelatedNoise)} = \left|\mathrm{CV}_{(B[\mathrm{model}])} - \mathrm{CV}_{(B[\mathrm{data}])}\right| + \left|\mathrm{CV}_{(E[\mathrm{model}])} - \mathrm{CV}_{(E[\mathrm{data}])}\right| + \left|\mathrm{CV}_{(L[\mathrm{model}])} - \mathrm{CV}_{(L[\mathrm{data}])}\right| + \left|\mathrm{CV}_{(E/L[\mathrm{model}])} - \mathrm{CV}_{(E/L[\mathrm{data}])}\right|.$$

Next, the total cost function was calculated which is the sum of the two normalized cost functions matrices. The correlated and uncorrelated noise combination returning minimum value of the total cost function (Fig 5C and 5D) was used for all further analysis. For illustrative purposes, the cost functions for all combinations of correlated and uncorrelated noise in Figure 5C and 5D were normalized to the highest cost value of the simple ensemble model (Section IV.C). It can be seen that the cluster modeling

approach robustly explains the data over a broad range of noise levels, and is superior of an alternative modeling approach (Section IV.C). The best-fit is observed for the following noise levels: noise1 = std(correlated) = 0.22 and noise2 = std(uncorrelated) = 0.15. These noise levels were used for further analysis (FB knockout: see below), and to plot the comparisons between signaling features in the model and in the experimental data (Fig 5E).

IV.C Simulation of a complete cell population (simple ensemble model approach)

Previous modeling studies of single-cell heterogeneity did not use the cluster fitting approach described above, but employed a simpler method (termed "simple ensemble approach" hereafter) in which an ensemble of single cells was simulated by direct sampling the signaling protein concentrations from log-normal distributions around the population-average model fit. This essentially corresponds to the modeling framework described in sections IV.B, but leaving out the cluster fitting step. We asked whether our cluster fitting approach is advantageous when compared to the simple ensemble modeling approach.

Cell-to-cell variability was introduced into simple ensemble simulations of 10,000 single cells by sampling the protein concentrations and lumped parameters in Appendix Table S7 from log-normal distributions around their population average value (Appendix Table S5A). The ligand degradation by each single cell was again considered to be negligible (by increasing the medium volume), and a ligand degradation time course corresponding to the population-average model was imposed in each simulation (as described in Section IV.A). As before, a noise titration was performed, in which we systematically tested different levels of correlated and uncorrelated noise in the protein fluctuations and asked which noise level best reproduces the experimental measured heterogeneity. The 2D grid for scanning the two noise contributions was the same as for the cluster modeling approach (Section IV.B), and it was again assumed that all signaling protein concentrations exhibit the same degree of variability around their mean value. The optimal combination of correlated and uncorrelated noise contributions was assessed by the cost function described in Section IV.B.

The heat maps in Figure 5C and 5D shows the value of this cost function for each combination of correlated and uncorrelated noise levels. The simpler ensemble modeling performs less well than the cluster fitting in explaining the data, as judged by two criteria: (i) the minimal value of the cost function is larger for the simpler approach

than for the cluster fitting approach; (ii) the simpler approach is less robust, as a low value of the cost function requires fine-tuning of the two noise contributions, which is not the case for the cluster fitting approach. We therefore decided to predict novel experimental conditions using the cluster fitting approach (see Section IV.B).

IV.D Quantitative analysis of signaling dynamics in SMAD7 knockout cells

Having established a quantitative model of signaling heterogeneity (Section IV.B), we asked which signaling protein fluctuations may underlie heterogeneous signaling. In this section, it will be explained how we identified transcriptional negative feedback as a major determinant of decomposition of the cell population into distinct signaling classes and how we then quantitatively compared model simulations for feedback-depleted cells to corresponding experimental SMAD7 knockout measurements.

Identification of transcriptional negative feedback as a major determinant of signaling heterogeneity: We sought to determine in an unbiased way which signaling protein concentration or kinetic parameter fluctuations can explain best the decomposition into distinct dynamic signaling classes. To this end, we analyzed the fitting results of the six subpopulation models (Appendix Table S7), and assessed patterns in protein/parameter composition using information theoretical approaches (see below). To ensure the robustness of these results we did not restrict this analysis to the best-fit parameter sets (Appendix Table S7), but analyzed 30 independent fits with comparable log likelihood values that we had obtained by multi-start optimization (see Section 'Optimization').

Information theory indicates that the negative feedback is the main driver of signaling heterogeneity*:* To identify parameters of the mathematical model that contribute most do decomposing SMAD responses into distinct signaling classes, we make use of concepts from information theory. The model includes parameters $p_{1..n}$ with distinct values b for the m different signaling classes (see Appendix Table S7 except index_ active_Rec_internalize, index_k_out_1_relative_speed, index_k_out_2_relative_speed as these parameters dependent on the value of k_in_R1). The information content of the corresponding vector $B_p = \{b_{p,1}, \dots, b_{p,m}\}$ can be measured as entropy. Entropy (Shannon & E., 2001) quantifies uncertainty in the information source and hence gives a measure for the uniformity of a probability

distribution defined by the normalized vector $\tilde{B} = B_P/\Sigma(B_{P)}$. To measure whether the initial values of a parameter differ among the clusters we compute the entropy for each parameter as $H_P = -\sum_{i=1}^{m} \tilde{B}_P(i) \cdot log_2\left(\tilde{B}_P(i)\right)$ (Figure 6D) . If all values of a parameter among the clusters are very similar, the entropy is high and vice versa the more heterogeneous a parameter is among the different clusters the lower is the measured entropy. To prevent bias from parameter fitting, we calculated the distribution of entropy values for 30 independent fits of the initial population model to the individual signaling classes.

For all the independent model parameters with comparable goodness-of-fit, it appears that the most discriminating factor among clusters is the parameter controlling production rate of the feedback protein (Fig 6D). Thus, the model predicted that a perturbation of this feedback element efficiently affects the distribution of cells into clusters and their dynamics.

Quantitative comparison of feedback-depleted model simulations to SMAD7 knockout data: One important and well-established transcriptional feedback regulator of the SMAD signaling pathway is SMAD7 (Legewie *et al*, 2008; Nicklas & Saiz, 2013). To experimentally verify the impact of feedback on signaling dynamics, we generated mutant MCF10A-SMAD2-YFP cells in which both wild-type copies of SMAD7 were replaced by a truncated version of the protein that no longer interferes with TGFβ/SMAD signaling (see Material and Methods). This corresponds to a functional SMAD7 knockout, and at the population-average level we observed that this perturbation increased both the initial amplitude and late signaling plateau of SMAD2 nuclear translocation when compared to control (Figure EV5G).

TGFβ signaling is controlled by multiple transcriptional negative feedback loops (Legewie *et al*, 2008), making it unlikely that a full knockout of the generic feedback regulator (FB) in the model directly corresponds to the SMAD7 knockout experiment. We therefore tested the effect of different degrees of feedback depletion on the signaling dynamics in a simulated cell population and quantitatively compared these single-cell simulations to the SMAD7 knockout data to ensure an optimal match between model prediction and experiment. As described above (Section III.C Model simulation), we simulated the reduction of FB in the best-fit model by systematically lowering the FB mRNA synthesis term (FB_KD_index, Appendix Table S3). FB_KD_index = 1 corresponds

to the WT situation, whereas FB_KD_index = 0 corresponds to complete elimination of the feedback loop. We systematically varied FB_KD_index between 0.5 to 0.01 to the same extent in all the six clusters performed single-cell simulations for each of the values (as described in Section IV.B) to construct a population of 10.000 cells.

We again used clustering to quantitatively compare FB-depleted single-cell simulations and SMAD7 knockout data. Single-cell SMAD7 knockout measurements and simulations were mapped onto the clusters obtained from original titration dataset (as descripted in Section II.H). This allows the assignment of all artificial and measured SMAD7 knockout single cell trajectories to the empirical signaling classes identified in the original titration dataset. Next, we compared the cluster distribution between model and data for different strengths of feedback in the simulations. For this, we subtracted the simulated and measured number of cells in each cluster and used the summed-up difference over all clusters at 100 pM TGFb as a measure of model-data divergence. We found that for a feedback strength of ~70% (FB_KD_index = 0.3), the experimental effect of Smad7 KO on cluster redistribution was the closest to model (Figure 6F). The apparently remaining feedback in the model suggests that the TGFβ signaling pathway is not controlled by a single FB, but by multiple redundant ones, with SMAD7 accounting for the majority of feedback. Using the same degree of feedback depletion and by adjusting the global ligand degradation dynamics (Section IV.A), we can also successfully predict alterations of the SMAD2 dynamics at lower TGFβ concentrations (Figure 6E and G). These predictions were robust against parameter uncertainties, as we obtained similar results when applying the same degree of feedback depletion assuming the same contributions of correlated and uncorrelated protein concentration noise to each of the 30 independent fits to the signaling class dynamics (Figure EV5D). Since we use the same samples of the protein concentrations and parameters at different TGFβ concentrations in wildtype vs. SMAD7 knockout simulations, we can use our mapping approach to determine transition from one cluster to another with changing experimental conditions (Figure 6B) or altered knock-out strength (Figure 6C). Taken together, our results indicate that the extent of SMAD7-mediated feedback in individual cells partly determines to which cluster a cell belongs, thereby playing an important role in controlling the heterogeneous dynamics of SMAD2 nuclear translocation.

V. References

Abe M, Harpel JG, Metz CN, Nunes I, Loskutoff DJ & Rifkin DB (1994) An Assay for Transforming Growth Factor-β Using Cells Transfected with a Plasminogen Activator Inhibitor-1 Promoter-Luciferase Construct. *Anal. Biochem.* **216:** 276–284

Bensaude O (2011) Inhibiting eukaryotic transcription. Which compound to choose? How to evaluate its activity? *Transcription* **2:** 103–108

Berndt DJ & Clifford J (1994) Using dynamic time warping to find patterns in time series. *Proc. 3rd Int. Conf. Knowl. Discov. Data Min.*: 359–370

Boesen CC, Radaev S, Motyka SA, Patamawenu A & Sun PD (2002) The 1.1 Å Crystal Structure of Human TGF-β Type II Receptor Ligand Binding Domain. *Structure* **10:** 913–919

Carpenter AE, Jones TR, Lamprecht MR, Clarke C, Kang IH, Friman O, Guertin DA, Chang JH, Lindquist RA, Moffat J, Golland P & Sabatini DM (2006) CellProfiler: image analysis software for identifying and quantifying cell phenotypes. *Genome Biol.* **7:** R100

Chacko BM, Qin BY, Tiwari A, Shi G, Lam S, Hayward LJ, de Caestecker M & Lin K (2004) Structural Basis of Heteromeric Smad Protein Assembly in TGF-β Signaling. *Mol. Cell* **15:** 813–823

Chen X & Murphy RF (2005) Objective clustering of proteins based on subcellular location patterns. *J Biomed Biotechnol* **2005 (2):** 87-95

Cohen AA, Geva-Zatorsky N, Eden E, Frenkel-Morgenstern M, Issaeva I, Sigal A, Milo R, Cohen-Saidon C, Liron Y, Kam Z, Cohen L, Danon T, Perzov N & Alon U (2008) Dynamic proteomics of individual cancer cells in response to a drug. *Science* **322:** 1511-1515

Dai F, Lin X, Chang C & Feng X-H (2009) Nuclear Export of Smad2 and Smad3 by RanBP3 Facilitates Termination of TGF-β Signaling. *Dev. Cell* **16:** 345–357

Dijke P ten, Nakao A, Afrakhte M, Morn A, Nakayama T, Christian JL, Heuchel R, Itoh S, Kawabata M, Heldin N-E & Heldin C-H (1997) Identification of Smad7, a TGF|[beta]|-inducible antagonist of TGF-|[beta]| signalling. *Nature* **389:** 631–635

Groß W (1915) Grundzüge der Mengenlehre. *Monatshefte für Math. und Phys.* **26:** A34–A35

Di Guglielmo GM, Le Roy C, Goodfellow AF & Wrana JL (2003) Distinct endocytic pathways regulate TGF-β receptor signalling and turnover. *Nat. Cell Biol.* **5:** 410–421

Handl J, Knowles J & Kell DB (2005) Computational cluster validation in post-genomic data analysis. *Bioinformatics* **21:** 3201–3212

Inman GJ & Hill CS (2002) Stoichiometry of active smad-transcription factor complexes on DNA. *J. Biol. Chem.* **277:** 51008–16

Jeschke M, Baumgärtner S, Legewie S, Bergmann A & Goncalves M (2013) Determinants of Cell-to-Cell Variability in Protein Kinase Signaling. *PLoS Comput. Biol.* **9:** e1003357

Kallenberger SM, Beaudouin J, Claus J, Fischer C, Sorger PK, Legewie S & Eils R (2014) Intra- and Interdimeric Caspase-8 Self-Cleavage Controls Strength and Timing of CD95-Induced Apoptosis. *Sci. Signal.* **7:** ra23-ra23

Kavsak P, Rasmussen RK, Causing CG, Bonni S, Zhu H, Thomsen GH & Wrana JL (2000) Smad7 Binds to Smurf2 to Form an E3 Ubiquitin Ligase that Targets the TGFβ Receptor for Degradation. *Mol. Cell* **6:** 1365–1375

Keogh EJ & Pazzani MJ (2000) Scaling up dynamic time warping for datamining applications. In *Proceedings of the sixth ACM SIGKDD international conference on Knowledge discovery and data mining - KDD '00* pp 285–289. New York, New York,

USA: ACM Press
Legewie S, Herzel H, Westerhoff H V & Blüthgen N (2008) Recurrent design patterns in the feedback regulation of the mammalian signalling network. *Mol. Syst. Biol.* **4 (1):** 190
Lipkin BS (1970) Picture Processing and Psychopictorics. Elsevier Science
López-Casillas F, Wrana JL & Massagué J (1993) Betaglycan presents ligand to the TGFβ signaling receptor. *Cell* **73:** 1435–1444
Massagué J & Chen YG (2000) Controlling TGF-beta signaling. *Genes Dev.* **14:** 627–44
Mitchell H, Choudhury A, Pagano RE & Leof EB (2004) Ligand-dependent and -independent transforming growth factor-beta receptor recycling regulated by clathrin-mediated endocytosis and Rab11. *Mol. Biol. Cell* **15:** 4166–78
Nicklas D & Saiz L (2013) Computational modelling of Smad-mediated negative feedback and crosstalk in the TGF-β superfamily network. *J. R. Soc. Interface* **10:** 20130363
Nishi H, Hashimoto K & Panchenko AR (2011) Phosphorylation in Protein-Protein Binding: Effect on Stability and Function. *Structure* **19:** 1807–1815
Raue A, Schilling M, Bachmann J, Matteson A, Schelke M, Kaschek D, Hug S, Kreutz C, Harms BD, Theis FJ, Klingmüller U & Timmer J (2013) Lessons Learned from Quantitative Dynamical Modeling in Systems Biology. *PLoS ONE* **8**(9)**:** e74335
Raue A, Steiert B, Schelker M, Kreutz C, Maiwald T, Hass H, Vanlier J, Tönsing C, Adlung L, Engesser R, Mader W, Heinemann T, Hasenauer J, Schilling M, Höfer T, Klipp E, Theis F, Klingmüller U, Schöberl B & Timmer J (2015) Data2Dynamics: a modeling environment tailored to parameter estimation in dynamical systems. *Bioinformatics* **31:** 3558–3560
Razani B, Zhang XL, Bitzer M, von Gersdorff G, Böttinger EP & Lisanti MP (2001) Caveolin-1 regulates transforming growth factor (TGF)-beta/SMAD signaling through an interaction with the TGF-beta type I receptor. *J. Biol. Chem.* **276:** 6727–38
Rhee A, Cheong R & Levchenko A (2014) Noise decomposition of intracellular biochemical signaling networks using nonequivalent reporters. *Proc. Natl. Acad. Sci.* **111:** 17330–17335
Rousseeuw PJ (1987) Silhouettes: A graphical aid to the interpretation and validation of cluster analysis. *J. Comput. Appl. Math.* **20:** 53–65
Le Roy C & Wrana JL (2005) Clathrin- and non-clathrin-mediated endocytic regulation of cell signalling. *Nat. Rev. Mol. Cell Biol.* **6:** 112–126
Sakoe H & Chiba S (1978) Dynamic programming algorithm optimization for spoken word recognition. *IEEE Trans. Acoust.* **26:** 43–49
Schmierer B & Hill CS (2005) Kinetic analysis of Smad nucleocytoplasmic shuttling reveals a mechanism for transforming growth factor beta-dependent nuclear accumulation of Smads. *Mol. Cell. Biol.* **25:** 9845–58
Schmierer B & Hill CS (2007) TGFβ–SMAD signal transduction: molecular specificity and functional flexibility. *Nat. Rev. Mol. Cell Biol.* **8:** 970–982
Schmierer B, Tournier AL, Bates PA & Hill CS (2008) Mathematical modeling identifies Smad nucleocytoplasmic shuttling as a dynamic signal-interpreting system. *Proc. Natl. Acad. Sci. U. S. A.* **105:** 6608–13
Shannon CE (2001) A mathematical theory of communication. *ACM SIGMOBILE Mob. Comput. Commun. Rev.* **5** (1)**:** 3-55
Shi Y & Massagué J (2003) Mechanisms of TGF-β Signaling from Cell Membrane to the Nucleus. *Cell* **113:** 685–700
Sigal A, Milo R, Cohen A, Geva-Zatorsky N, Klein Y, Alaluf I, Swerdlin N, Perzov N, Danon T & Liron Y (2006) Dynamic proteomics in individual human cells uncovers

widespread cell-cycle dependence of nuclear proteins. *Nat Methods* **3** (7)**:** 525-531
Spencer SL, Sorger PK, Rehm M, Lawrence DA, Pitti RM, Lancaster K, Lee D, Goetz M von, Yee SF, Totpal K & al. et (2011) Measuring and modeling apoptosis in single cells. *Cell* **144:** 926–39
Sugar CA & James GM (2003) Finding the Number of Clusters in a Dataset. *J. Am. Stat. Assoc.* **98:** 750–763
Swain PS, Elowitz MB & Siggia ED (2002) Intrinsic and extrinsic contributions to stochasticity in gene expression. *Proc. Natl. Acad. Sci. U. S. A.* **99:** 12795–800
Vizán P, Miller DSJ, Gori I, Das D, Schmierer B & Hill CS (2013) Controlling Long-Term Signaling: Receptor Dynamics Determine Attenuation and Refractory Behavior of the TGF-β Pathway. *Sci. Signal.* **6** (305)**:** ra106
Ward JH (1963) Hierarchical Grouping to Optimize an Objective Function. *J. Am. Stat. Assoc.* **58:** 236–244
Wegner K, Bachmann A, Schad J-U, Lucarelli P, Sahle S, Nickel P, Meyer C, Klingmüller U, Dooley S & Kummer U (2012) Dynamics and feedback loops in the transforming growth factor β signaling pathway. *Biophys. Chem.* **162:** 22–34
Wu J-W, Hu M, Chai J, Seoane J, Huse M, Li C, Rigotti DJ, Kyin S, Muir TW, Fairman R, Massagué J & Shi Y (2001) Crystal Structure of a Phosphorylated Smad2: Recognition of Phosphoserine by the MH2 Domain and Insights on Smad Function in TGF-β Signaling. *Mol. Cell* **8:** 1277–1289
Xu P, Liu J & Derynck R (2012) Post-translational regulation of TGF-β receptor and Smad signaling. *FEBS Lett.* **586:** 1871–1884
Zi Z, Feng Z, Chapnick DA, Dahl M, Deng D, Klipp E, Moustakas A & Liu X (2011) Quantitative analysis of transient and sustained transforming growth factor-β signaling dynamics. *Mol. Syst. Biol.* **7:**492
