# Supplementary material for: Cell‐specific responses to the cytokine TGFβ are determined by variability in protein levels
Source: Mol Syst Biol. 2018 Jan 25;14(1):e7733. doi: 10.15252/msb.20177733 (PMC5787704; doi:10.15252/msb.20177733)
Supplement: Supplementary file 2 — Expanded View Figures PDF [file MSB-14-e7733-s002.pdf]

Expanded View Figures

Figure EV1. Dynamics and variability of SMAD2 signaling in single cells.

A Western blot analysis of SMAD2 activation in SMAD2-YFP reporter and parental MCF10A cells. Cells were stimulated with 100 pM TGFβ1 and SMAD2 phosphorylation was analyzed at indicated time points. GAPDH was used as a loading control. Independent experiments were quantified and normalized to maximum values. Error bars indicate standard deviation of biological repeats (n = 4). Note that phosphorylated YFP-SMAD2 was at background levels at 0.25 h presumably due to lower expression levels.

B Expression of SMAD target genes in parental and SMAD2 reporter cell lines. Expression kinetics of the SMAD target genes SMAD7, SnoN, and PAI-1 upon 100 pM TGFβ stimulation were measured by qPCR in the indicated cell lines. β-Actin was used as an internal control. Error bars indicate standard deviation of technical triplicates.

C Live-cell time-lapse microscopy images of H2B-CFP expression in MCF10A cells following treatment with 100 pM TGFβ. The same detail as in Fig 1D is shown. White circle indicates the segmented nucleus, and the estimated cytoplasmic area is represented by red annuli.

D–F The cell indicated in Fig 1D was tracked over 24 h. The mean nuclear (D) and cytoplasmic (E) fluorescence intensity of SMAD2-YFP as well as the nuclear fluorescence intensity of H2B-CFP (F) were measured upon 100 pM TGFβ stimulation.

G Reproducibility of SMAD2 translocation measurements. Median SMAD2-YFP ratios (solid lines) of cells plated in three independent glass bottom plates stimulated with 100 pM TGFβ at the same day and tracked over 24 h (biological triplicates). Shaded areas indicate 25th and 75th percentiles. See Appendix Table S1 for number of cells analyzed.

H Correlation between endogenously expressed SMAD2 and transgenic YFP-SMAD2. In the same individual SMAD2 reporter cells treated with 100 pM TGFβ for 1.5 h, nuclear endogenous SMAD2 was measured by immunofluorescence and compared to the nuclear fluorescence intensity from YFP-SMAD2. Both measures were highly correlated (Pearson's correlation, n = 7,300).

I Comparison of endogenous SMAD2 activation and SMAD2-YFP translocation. The nuc/cyt ratio of SMAD2-YFP upon 100 pM TGFβ stimulation was measured in reporter cells by time-lapse microscopy at the indicated time points (blue); phosphorylation of endogenous SMAD2 was measured in parental MCF10A cells by immunofluorescence (IF, red) under the same conditions. Data were normalized by minimum subtraction and division through the overall maximum. White and black dots indicate medians; boxes include data between the 25th and 75th percentiles; whiskers extend to maximum values within 1.5× the interquartile range; colored dots represent outliers. See Appendix Table S1 for number of cells analyzed.

© 2018 The Authors

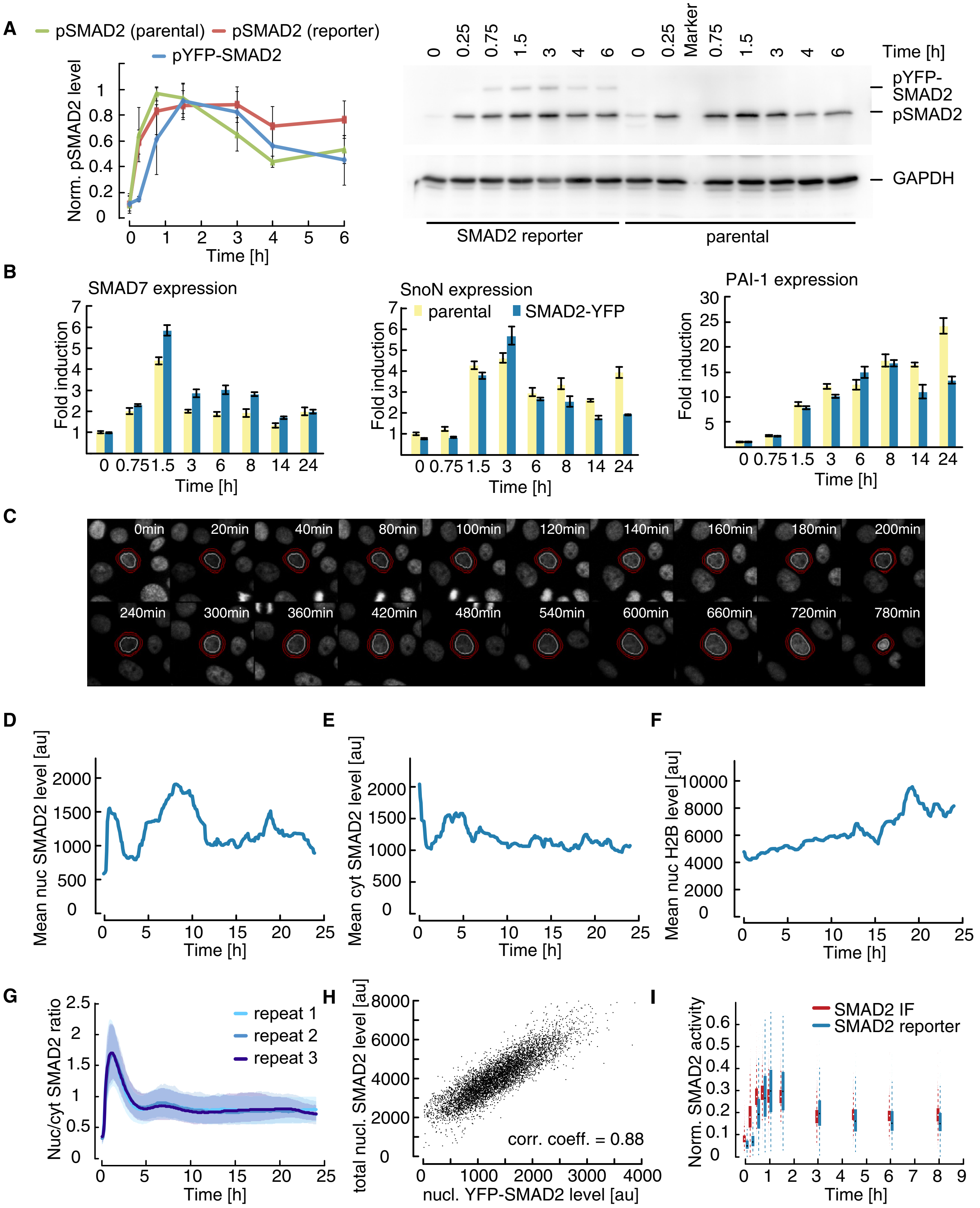
A
pSMAD2 (parental)
pSMAD2 (reporter)
pYFP-SMAD2
Norm. pSMAD2 level
Time [h]
0 0.25 0.75 1.5 3 4 6 0 0.25 Marker 0.75 1.5 3 4 6
Time [h]
pYFP-SMAD2
pSMAD2
GAPDH
SMAD2 reporter
parental
B
SMAD7 expression
SnoN expression
PAI-1 expression
parental
SMAD2-YFP
Fold induction
Time [h]
C
0min 20min 40min 80min 100min 120min 140min 160min 180min 200min
240min 300min 360min 420min 480min 540min 600min 660min 720min 780min
D
Mean nuc SMAD2 level [au]
Time [h]
E
Mean cyt SMAD2 level [au]
Time [h]
F
Mean nuc H2B level [au]
Time [h]
G
Nuc/cyt SMAD2 ratio
repeat 1
repeat 2
repeat 3
Time [h]
H
total nucl. SMAD2 level [au]
corr. coeff. = 0.88
nucl. YFP-SMAD2 level [au]
I
Norm. SMAD2 activity
SMAD2 IF
SMAD2 reporter
Time [h]

Figure EV1.

Figure EV2. Clustering heterogeneous SMAD translocation dynamics using dynamic time warping.

A, B Dose-dependency of SMAD target gene expression. Parental MCF10A cells were stimulated with varying concentrations of TGFβ and SMAD7 (A) and PAI-1 (B) expression was measured by qPCR at indicated time points. Error bars indicate standard deviation of technical triplicates.

C Illustrative comparison of Euclidian distance and dynamic time warping (DTW). In the left panel, three single-cell trajectories (red, blue, and black) with similar Euclidian distances are shown (see table). DTW performs a non-linear alignment in time (middle panel) that compensates the temporal shift of the peaks in the red and blue trajectory (cyan lines). This leads to a lower DTW distance score compared to the DTW distance between the red and black or blue and black trajectories, which remain almost unchanged (see table).

D Dissimilarity matrix calculated pair-wise by cDTW of single-cell trajectories treated with varying TGFβ doses. Strength of the TGFβ stimulation increases from top left to bottom right.

E Heat map of single-cell time courses sorted according to hierarchical clustering. The corresponding dendrogram is shown on top.

F Optimal number of clusters. For different cluster numbers, jump size is calculated using sum of square errors of cDTW scores as a measure of intra-cluster dispersion. Jump size reaches maxima at three and six clusters, indicating that these are good choices for cluster number.

G–J Direction-resolved analysis of cell motility in TGFβ-stimulated cells. The angle and distance of each cell movement were determined and averaged for 0–30 h (upper panel) and 30–60 h (lower panel) after stimulation with varying concentrations of TGFβ (see Appendix II.F for details). Cells are grouped according to stimulation levels (G) or signaling classes (H). Changes in cell motility are more pronounced at later time points after stimulation. Unidirectional movements (angle = 0) of TGFβ-stimulated cells 30–60 h after treatment were normalized by the mean movement of unstimulated cells in the same time period and analyzed according to stimulus level (I) or signaling classes (J). Changes in cell motility are express as median fold change; error bars indicate 95% confidence intervals from permutation testing. Signaling dynamics allow better stratification of cellular outcomes compared to stimulus levels. See Appendix Table S1 for number of cells analyzed.

▶

© 2018 The Authors

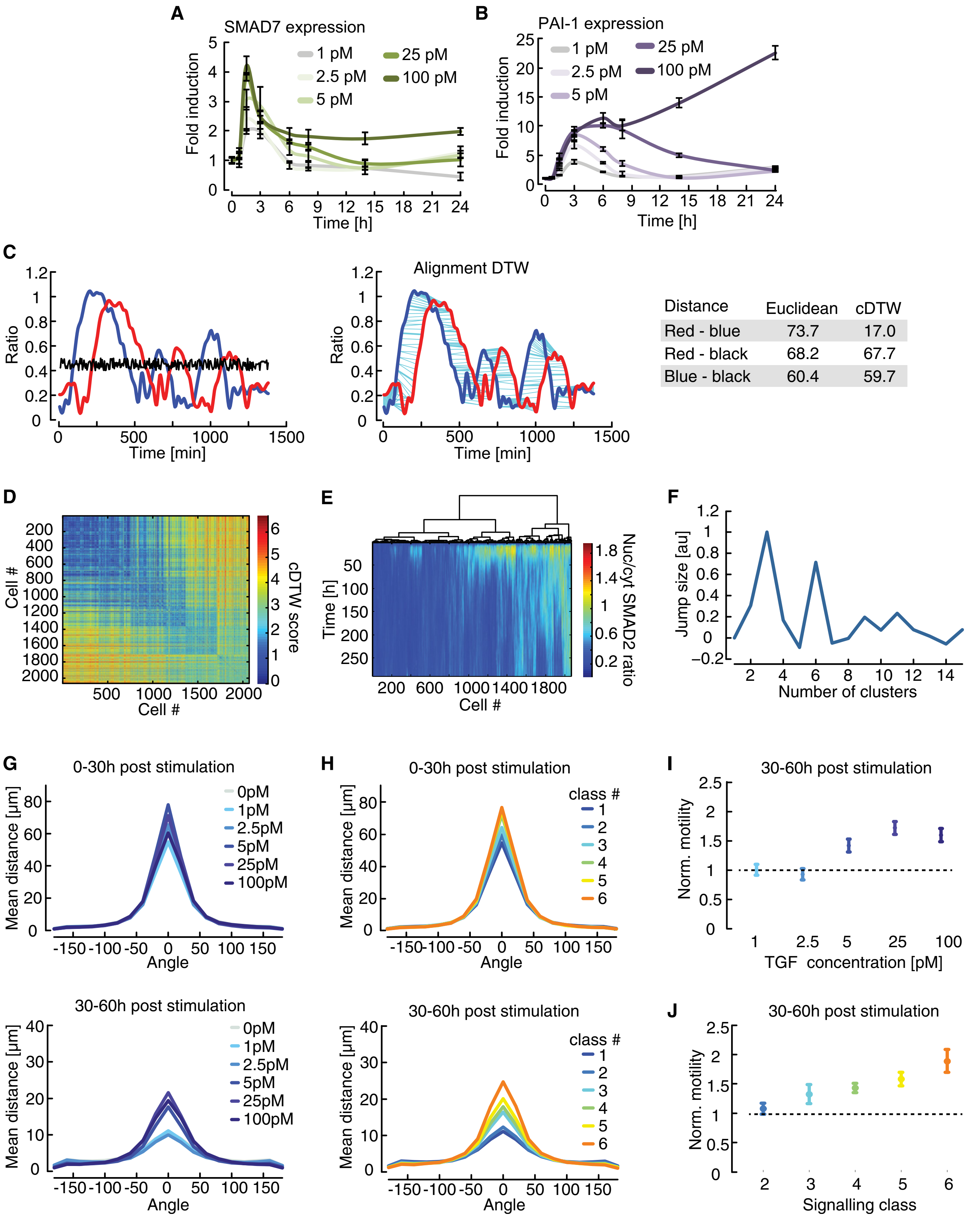
A
SMAD7 expression
1 pM
2.5 pM
5 pM
25 pM
100 pM
Fold induction
Time [h]
B
PAI-1 expression
1 pM
2.5 pM
5 pM
25 pM
100 pM
Fold induction
Time [h]
C
Alignment DTW
Ratio
Time [min]
Distance Euclidean cDTW
Red - blue 73.7 17.0
Red - black 68.2 67.7
Blue - black 60.4 59.7
D
Cell #
cDTW score
E
Time [h]
Cell #
Nuc/cyt SMAD2 ratio
F
Jump size [au]
Number of clusters
G
0-30h post stimulation
0pM
1pM
2.5pM
5pM
25pM
100pM
Mean distance [µm]
Angle
30-60h post stimulation
H
0-30h post stimulation
class #
30-60h post stimulation
I
30-60h post stimulation
Norm. motility
TGF concentration [pM]
J
30-60h post stimulation
Norm. motility
Signalling class

Figure EV2.

Figure EV3. Determining causes of heterogeneity in SMAD signaling.

A Time-resolved analysis of SMAD2 nuclear/cytoplasmic localization before and after stimulation with varying concentrations of TGFβ. Cells were imaged for 24 h before and 12 h after stimulation. Median nuc/cyt SMAD2 ratios for indicated stimulation levels are shown. See Appendix Table S1 for number of cells analyzed.

B Heat map of SMAD2 translocation in individual cells imaged for 24 h before stimulation with 25 pM or 5 pM TGFβ (similar to Fig 3A). Each horizontal line represents a single cell. Cells were sorted either by the time of the last division before stimulation (left) or by the amplitude of their response (right). Obviously, the time of last division and signaling responses are not correlated. See Appendix Table S1 for number of cells analyzed.

C Time of last cell division before stimulus for each signaling class after stimulation with the indicated concentrations of TGFβ. Single-cell time courses were mapped onto the previously observed signaling classes (Fig 2C; see Appendix II.H). Distributions are overlapping; no significant trend in cell division time is observable. White lines indicate median; boxes include data between the 25th and the 75th percentiles; whiskers extend to maximum values within 1.5× the interquartile range; crosses represent outliers. See Appendix Table S1 for number of cells analyzed.

D SMAD2 response in G2 arrested cells. Cells were arrested in G2 using the CDK1 inhibitor RO3306, stimulated with 100 pM TGFβ and followed for 24 h by live-cell imaging. No difference to control cells treated with vehicle could be observed. Solid lines indicate median nuc/cyt SMAD2 ratios, shaded areas 25th to 75th percentiles. See Appendix Table S1 for number of cells analyzed.

E Measuring local cell density by live-cell imaging. Local cell density is measured in a 200-px radius around each cell for each time point by applying a bell-shaped kernel to obtain a weighted sum of all neighboring cells (left). The resulting density scores are demonstrated using a randomly chosen time point. Red circles indicate the centroid of cells identified. Cells highlighted by blue circles were successfully tracked for the time of the experiment. Warmer colors indicate higher density scores.

F Cell density before stimulation shown for each signaling class observed in response to stimulation with the indicated concentrations of TGFβ. Distributions of density scores are overlapping; no significant trend in cell density is observable. White lines indicate median; boxes include data between the 25th and the 75th percentiles; whiskers extend to maximum values within 1.5× the interquartile range; crosses represent outliers. See Appendix Table S1 for number of cells analyzed.

G Quantifying the contribution of cell cycle state to heterogeneity in SMAD signaling. Mutual information between time of last cell division and nuc/cyt SMAD ratio after stimulation with varying doses of TGFβ was determined for each time point and normalized by the sum of entropies to calculate the fraction of heterogeneity in SMAD signaling that can be explained by cell cycle state (Appendix II.E).

H Quantifying the contribution of cell density to heterogeneity in SMAD signaling. Mutual information between cell density scores and nuc/cyt SMAD ratio after stimulation with varying doses of TGFβ was determined pair-wise for all combinations of time point and normalized by the sum of entropies to calculate the fraction of heterogeneity in SMAD signaling that can be explained by cell density at any time point. The corresponding heat map demonstrates that cell density provides only a minor contribution to heterogeneity.

 © 2018 The Authors

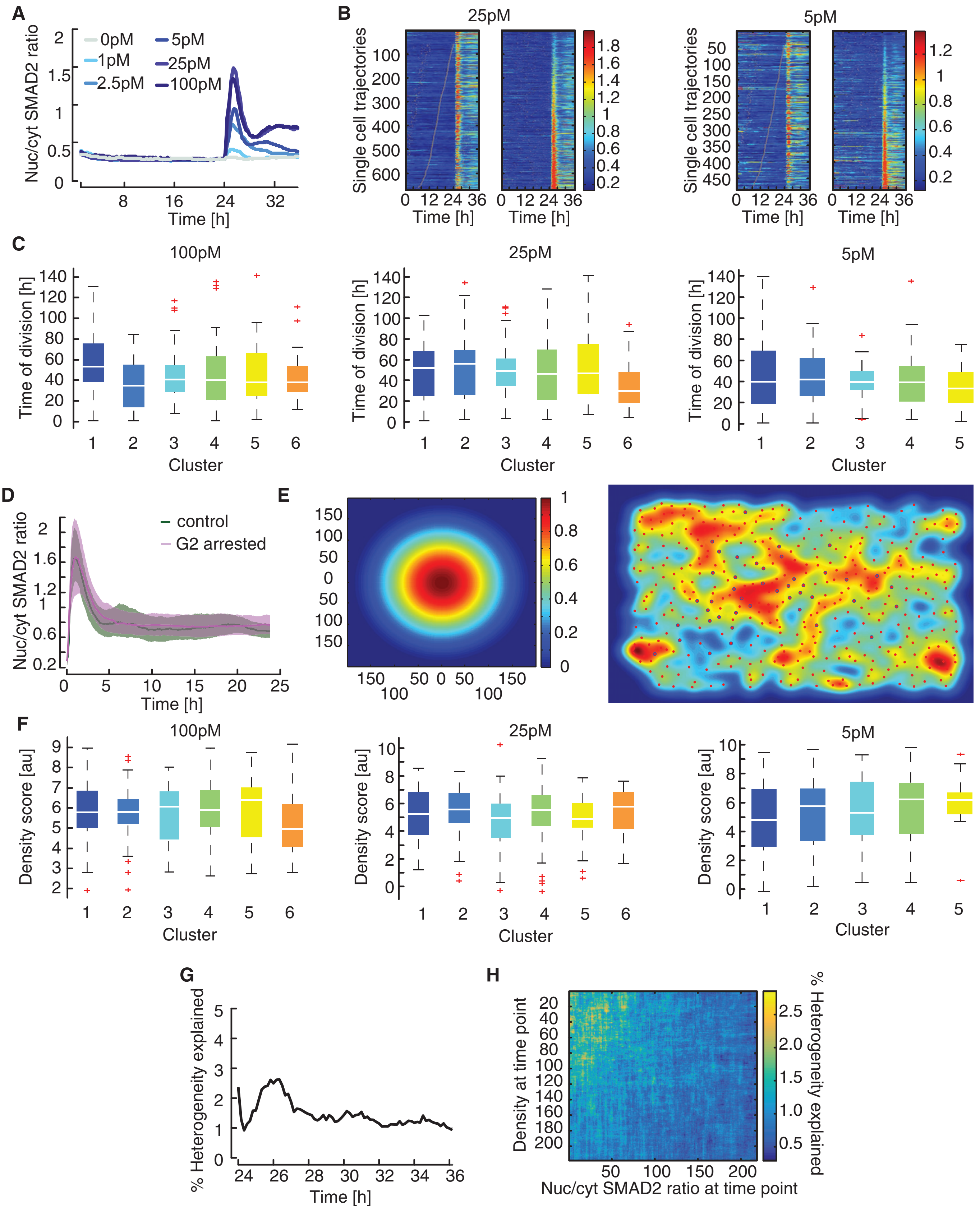
A
Nuc/cyt SMAD2 ratio
Time [h]
0pM
1pM
2.5pM
5pM
25pM
100pM
B
25pM
5pM
Single cell trajectories
Time [h]
C
100pM
25pM
5pM
Time of division [h]
Cluster
D
Nuc/cyt SMAD2 ratio
control
G2 arrested
Time [h]
E
F
100pM
25pM
5pM
Density score [au]
Cluster
G
% Heterogeneity explained
Time [h]
H
Density at time point
Nuc/cyt SMAD2 ratio at time point
% Heterogeneity explained

Figure EV3.

Figure EV4. Calibration of a population-average model.

A Model calibration with median SMAD4 translocation dynamics of cells stimulated with different TGFβ concentrations. Solid lines represent model fit, filled circles measured data. Experimental data points correspond to Appendix Fig S2J.

B, C Dynamics of total cellular TGFβ receptor protein levels upon stimulation with 100 pM TGFβ. Dynamics of TGFβR1 (B) and TGFβR2 (C) are shown as fold change relative to unstimulated cells. Solid red lines represent model fit and circles data measured by Western blot analysis. For each observation point, data spread from triplicates is shown. Gray area indicates measurement noise estimated by the fitting algorithm.

D Restimulation with 2.5 pM TGFβ. Stimulation was performed once at the beginning of the experiment (left), at the beginning and at 3 h (middle), or at the beginning and at 6 h (right) to replenish the ligand pool to its initial concentration. Solid lines represent model fits, and circles measured data points. Gray area indicates measurement noise estimated by the fitting algorithm.

E, F Model calibration with median SMAD2 and SMAD4 translocation dynamics upon receptor inhibition. Cells were stimulated with 100 pM TGFβ, TGFβRI activity was inhibited using the small-molecule receptor inhibitor SB431542 at the indicated time points (dashed vertical lines). Nuc/cyt SMAD2 (E) and SMAD4 (F) ratios were considered. Solid lines represent model fits, and circles or asterisks measured data. Experimental data points correspond to Fig 1G and Appendix Fig S2K. Gray area indicates measurement noise estimated by the fitting algorithm.

G Smad7 mRNA induction upon 25 pM and 100 pM TGFb stimulation. Solid lines represent model fits, and circles measured fold changes relative to unstimulated cells. Experimental data points correspond to Fig EV2A. Gray area indicates measurement noise estimated by the fitting algorithm.

H Model prediction of restimulation with 100 pM TGFβ at 3 h (dashed vertical line). Solid line represents model prediction, and asterisks (*) measured data.

 © 2018 The Authors

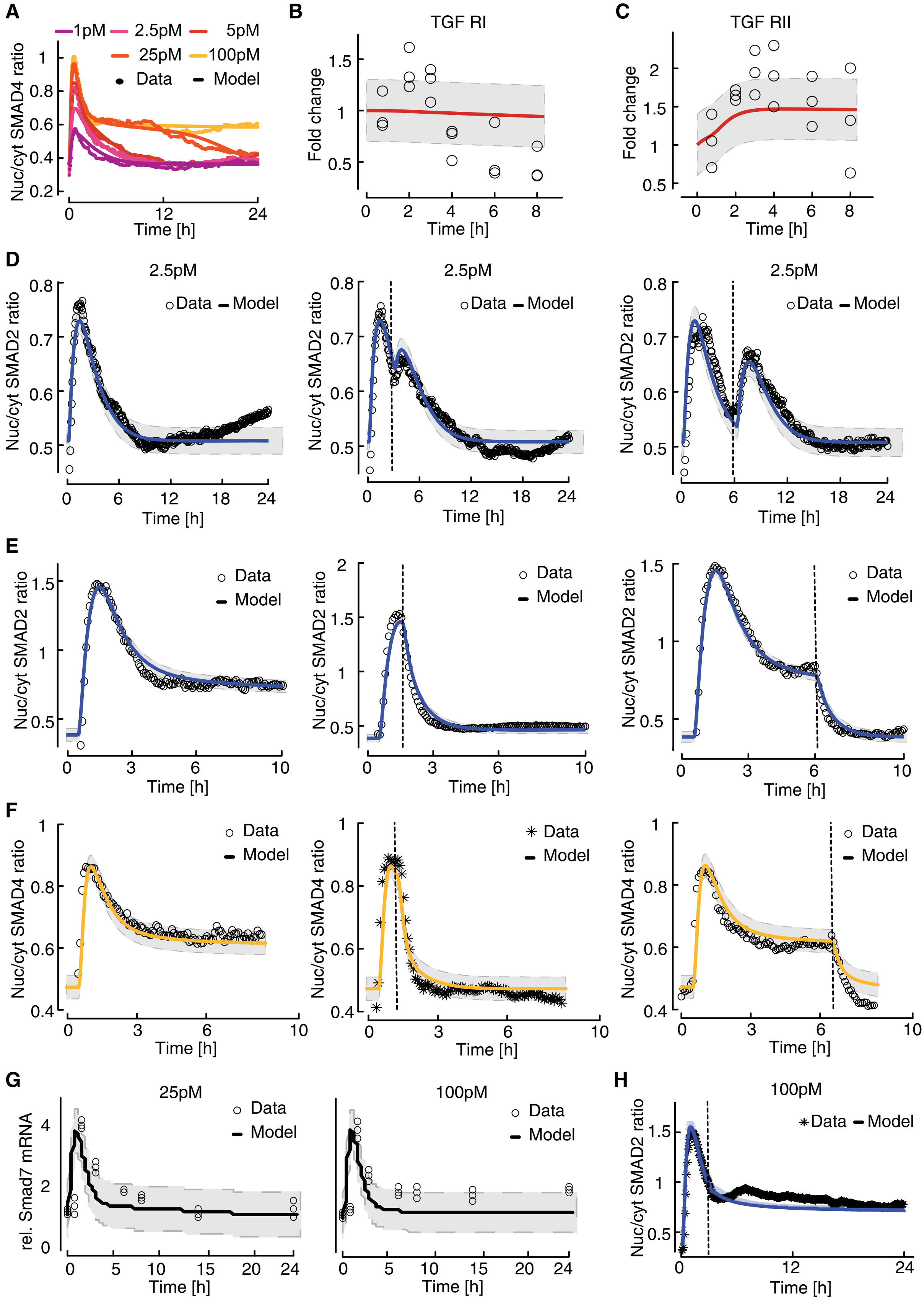
A
1pM
2.5pM
5pM
25pM
100pM
Data
Model
Nuc/cyt SMAD4 ratio
Time [h]
B
TGF RI
Fold change
Time [h]
C
TGF RII
Fold change
Time [h]
D
2.5pM
Data
Model
Nuc/cyt SMAD2 ratio
Time [h]
E
Data
Model
Nuc/cyt SMAD2 ratio
Time [h]
F
Data
Model
Nuc/cyt SMAD4 ratio
Time [h]
G
25pM
100pM
Data
Model
rel. Smad7 mRNA
Time [h]
H
100pM
Data
Model
Nuc/cyt SMAD2 ratio
Time [h]

Figure EV4.

Figure EV5. Modeling heterogeneous signaling dynamics predicts negative feedback as major determinant of signaling classes.

A Unimodal distribution of protein concentrations in artificial cell populations. Distributions of basal concentrations for TGFβR1, SMAD2, and SMAD4 are shown for a population of artificial cells assembled according to the observed proportion of signaling classes at 100 pM TGFβ using calibrated noise levels.

B Silhouette plots of artificial cells sorted according to TGFβ concentration (left panel) or mapped to experimentally observed signaling classes (right panel). Plots provide a graphical representation of how well the nuc/cyt SMAD2 ratios of each simulated cell corresponds to trajectories of other simulated cells in its own group. Positive silhouette scores indicate that SMAD2 responses are more similar to the own group, while negative scores signify that the corresponding trajectory is closer to any of the other groups. In general, signaling classes provide better separation than sorting according to stimulus levels.

C Transition between signaling classes depending on feedback strength. The response of a reassembled population of artificial cells to 5 and 25 pM TGFβ was simulated with reduced feedback expression as indicated and mapped to previously observed signaling classes (see Appendix II.H). Black lines and their thickness indicate the direction and extent of transitions between signaling classes. Transitions with a probability below 1% are excluded for better visualization.

D Robustness of model predictions concerning SMAD7 knock-out effect on distribution of signaling classes at 100 pM (top) and 5 pM (bottom) TGFβ, respectively. The simulated fraction of cells in each signaling class in wt and 30% feedback depleted cells is shown for 30 independent fits with similar goodness of fit obtained from local multistart optimization (see Appendix IV.D). The corresponding best-fit results are shown in Fig 6A and G. Artificial cell populations were generated by adding the same protein concentration noise as in the best-fit model (Fig 5C). White lines indicate median; boxes include data between the 25th and 75th percentiles; whiskers extend to maximum values within 1.5× the interquartile range; crosses represent outliers.

E Sequence of SMAD7 knock-out alleles. The indicated sequence (red) in the second exon of the SMAD7 gene was targeted by Cas9 in combination with a specific sgRNA. This led to deletions of 22 nt and 7 nt in the targeted alleles, causing frameshifts and non-sense mutations in the SMAD7 gene.

F Expression of SMAD7 mRNA in parental and knock-out cells. Basal SMAD7 mRNA levels were determined by qPCR in the indicated cell lines. SMAD7 mRNA containing a prematures stop codon is degraded in knock-out cells due to non-sense mediated decay. β-Actin was used as an internal control. Error bars indicate standard deviation of technical triplicates.

G Time-resolved analysis of SMAD2 nuclear/cytoplasmic translocation in the absence of SMAD7. Median nuc/cyt SMAD2 ratio of cells stimulated with varying concentrations of TGFβ over 24 h are shown for parental (left) and SMAD7 knock-out cells. See Appendix Table S1 for number of cells analyzed.

© 2018 The Authors

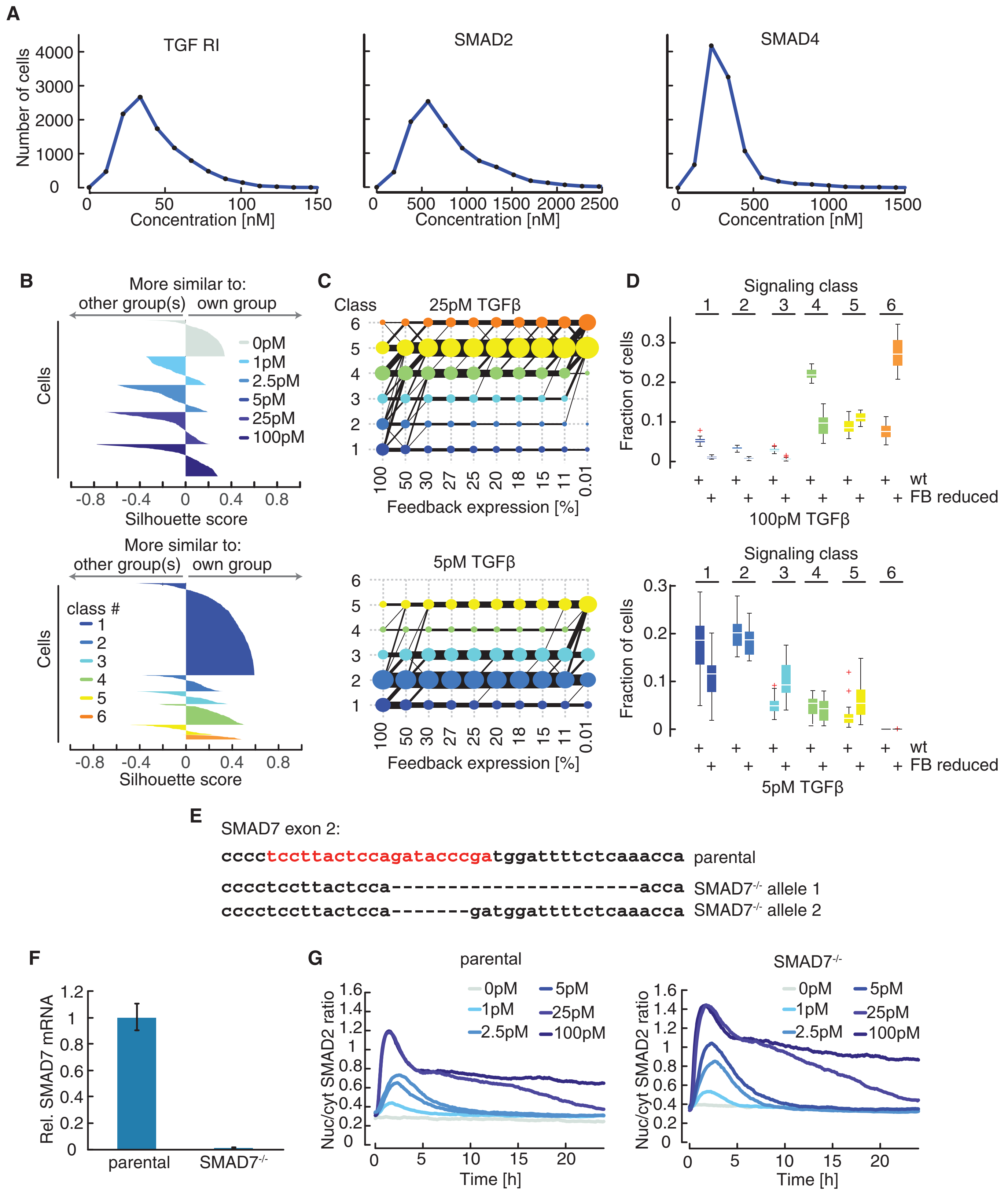
A
TGF RI
SMAD2
SMAD4
Number of cells
Concentration [nM]
B
More similar to:
other group(s)
own group
Cells
0pM
1pM
2.5pM
5pM
25pM
100pM
Silhouette score
class #
C
Class
25pM TGFβ
5pM TGFβ
Feedback expression [%]
D
Signaling class
Fraction of cells
wt
FB reduced
100pM TGFβ
5pM TGFβ
E
SMAD7 exon 2:
cccctccttactccagatacccgatggattttctcaaacca parental
cccctccttactcca----------------------acca SMAD7-/- allele 1
cccctccttactcca-------gatggattttctcaaacca SMAD7-/- allele 2
F
Rel. SMAD7 mRNA
parental
SMAD7-/-
G
parental
SMAD7-/-
Nuc/cyt SMAD2 ratio
Time [h]

Figure EV5.
